# Field-measured canopy height may not be as accurate and heritable as believed: evidence from advanced 3D sensing

**DOI:** 10.1186/s13007-023-01012-2

**Published:** 2023-04-02

**Authors:** Jingrong Zang, Shichao Jin, Songyin Zhang, Qing Li, Yue Mu, Ziyu Li, Shaochen Li, Xiao Wang, Yanjun Su, Dong Jiang

**Affiliations:** 1 Plant Phenomics Research Centre, Academy for Advanced Interdisciplinary Studies, Collaborative Innovation Centre for Modern Crop Production Co-Sponsored By Province and Ministry, College of Agriculture, Nanjing Agricultural University, Nanjing, 210095 China; 2 State Key Laboratory of Vegetation and Environmental Change, Institute of Botany, Chinese Academy of Sciences, Beijing, 100093 China

**Keywords:** Canopy height, Comparison, Field measurement, Terrestrial laser scanning, Backpack laser scanning, Gantry laser scanning, Digital aerial photogrammetry

## Abstract

Canopy height (CH) is an important trait for crop breeding and production. The rapid development of 3D sensing technologies shed new light on high-throughput height measurement. However, a systematic comparison of the accuracy and heritability of different 3D sensing technologies is seriously lacking. Moreover, it is questionable whether the field-measured height is as reliable as believed. This study uncovered these issues by comparing traditional height measurement with four advanced 3D sensing technologies, including terrestrial laser scanning (TLS), backpack laser scanning (BLS), gantry laser scanning (GLS), and digital aerial photogrammetry (DAP). A total of 1920 plots covering 120 varieties were selected for comparison. Cross-comparisons of different data sources were performed to evaluate their performances in CH estimation concerning different CH, leaf area index (LAI), and growth stage (GS) groups. Results showed that 1) All 3D sensing data sources had high correlations with field measurement (*r* > 0.82), while the correlations between different 3D sensing data sources were even better (*r* > 0.87). 2) The prediction accuracy between different data sources decreased in subgroups of CH, LAI, and GS. 3) Canopy height showed high heritability from all datasets, and 3D sensing datasets had even higher heritability (*H*^*2*^ = 0.79–0.89) than FM (field measurement) (*H*^*2*^ = 0.77). Finally, outliers of different datasets are analyzed. The results provide novel insights into different methods for canopy height measurement that may ensure the high-quality application of this important trait.

**Supplementary Information:**

The online version contains supplementary material available at 10.1186/s13007-023-01012-2.

## Highlights

- The effects of canopy height, leaf area index, and growth stage on the accurate monitoring of canopy height with different 3D sensors were systematically evaluated.
- Field-measured canopy height may not be as accurate as believed, especially in the plots with higher canopy height and at later growth stages.
- 3D sensing methods achieved higher heritable canopy height estimation than field measurement.

**Supplementary Information:**

The online version contains supplementary material available at 10.1186/s13007-023-01012-2.

## Introduction

Canopy height (CH) is an important and heritable agronomic trait for breeding and field management [34]**.** Breeders have paid much effort to selecting the ideal plant height to maximize light interception, increase yield [43], enhance logging resistance [57, 77], and facilitate mechanical harvesting. Agronomists often use CH to indicate the growth of other complicated and difficultly accessible traits, such as phenology [72], leaf area index (LAI) [11], and biomass [50]. Therefore, high-throughput and accurate evaluation (e.g., ensuring high heritability) of CH are critical for accelerating crop breeding and production.

Traditional CH estimation methods mainly use rulers by selecting a few representative positions within a canopy. Manual measurement is time-consuming, labor-intensive, tedious, and error-prone due to subjective selection and visual observation. However, it is still the most widely adopted way due to its visibility and reliability during the past decades. Recently, many studies have demonstrated CH can be efficiently acquired from advanced three-dimensional (3D) sensing techniques [28, 30, 43, 59, 68]. It brings us naturally to a fundamental and essential question: are 3D sensing techniques as accurate as field measurement?

Recent studies have explored the applicability of some mainstream 3D sensing techniques for CH measurements in agriculture, including LiDAR (light detection and management) and multi-view images [25]. LiDAR is an active sensing technology that records 3D structure information of objects by measuring the distance with the laser [8, 30]. LiDAR has many advantages, including (1) strong penetration ability that can characterize the inner structure of the canopy, (2) real and direct 3D characterization of an object without a complicated reconstruction process, and (3) insensitive to illumination. According to different mounting platforms, LiDAR systems used for crop height measurement mainly include terrestrial laser scanning (TLS) [14, 64], backpack laser scanning (BLS) [78], gantry laser scanning (GLS) [36, 61], and unmanned-aerial-vehicle laser scanning (ULS) [40, 54, 77]. In contrast to the active LiDAR sensing technologies, passive sensing-based methods (e.g., Multi-view images) can also measure 3D structure through methods like structure from motion (SFM) [3, 18, 45, 69]. Among the passive sensing-based techniques, digital aerial photogrammetry (DAP) is one of the most popular ways for field CH estimation due to its low cost, high efficiency, and high accuracy comparable to ULS [17, 21, 75, 76]. These 3D sensing techniques have been successfully applied to CH measurement, including the adoption of TLS for accurate height measurement of maize (R^2^ = 0.93) [64], cotton (R^2^ = 0.97) [60], rice (R^2^ = 0.91) [63], barley (R^2^ = 0.95), pea (R^2^ = 0.93), and bean (R^2^ = 0.91) [9], the use of BLS for efficient height measurement of large-scale wheat [78] and forest [22, 32, 58]; the exploration of ULS for estimating CH of sugar beet (R^2^ = 0.70), wheat (R^2^ = 0.78), and potato (R^2^ = 0.50) [24], and DAP for measuring corn CH (R^2^ = 0.78) [57]. In all, current studies demonstrated that TLS and BLS usually performed better than ULS and DAP due to their close range of sensing, and the accuracy of DAP was comparable to ULS (Additional file 1: Table S1).

In addition to the exploration of high estimation accuracy, more and more studies are attempting to explore the genetic bases (e.g., heritability) of high-throughput phenotype [56, 62, 73]. CH is a high heritability trait, as effective as yield [53]. Higher heritability indicates that the environment has less influence on the trait, and further describes the value of breeding [5, 48]. Several studies have already verified the potential of CH from many 3D sensing platforms, including the use of LiDAR [33, 68] and UAV imagery [67]. Interestingly, recent studies declared 3D sensing-derived CH showed better heritability than field measurement. For example, Madec et al. [43] proved high heritability values (*H*^*2*^ > 0.90) of CH derived from both LiDAR and DAP; Volpato et al. [67] compared the height heritability from UAV imagery (H^2^ = 0.71–0.97) and field measurement (H^2^ = 0.62–0.96) across four different growth stages (GS), which showed the UAV imagery had better heritability. These novel studies inspire us to rethink a questionable and challenging question: is field-measured CH as accurate and heritable as believed?

Some critical discussions about the accuracy of field-measured CH have been raised in recent years. On the one hand, field measurements are believed as accurate benchmarks. For example, Wang et al. [70] found the heights measured by the LIDAR-Lite v2, the Kinect v2 camera, ultrasonic, and the imaging array sensors had high correlations (r ≥ 0.90) with manual measurements. They believed that the errors among sensors and field measurements come from the sensor's error. On the other hand, more and more studies emphasized that there may be systematic errors in the ground truth values. For example, Maesano et al. [44] pointed out that LiDAR can detect more precise height differences than field measurement by comparing the accuracy of grass CH derived from ULS and field measurement. The inaccuracy of field-measured CH may be attributed to the variations of CH [68] and canopy structure [77]. Similarly, the heritability between 3D sensing and field measurement is also worth exploring.

This study aims to compare CH extraction accuracy and heritability from field measurements and four different proximal 3D sensing technologies, including TLS, BLS, GLS, and DAP in a wheat field of different varieties across different growth stages. Unlike previous studies, we make the following contributions: 1) systematically evaluating the accuracy of different data sources (TLS, BLS, GLS, DAP, and FM/field measurement) in estimating CH, 2) exploring the variations of height measurement accuracy concerning different CH, LAI, and the GS groups, 3) deciphering the error sources of CH measurement among different data sources, and 4) exploring the heritability of 3D sensing data sources in estimating CH.

## Materials and data collection

### Study area and experimental design

**Fig. 1:**
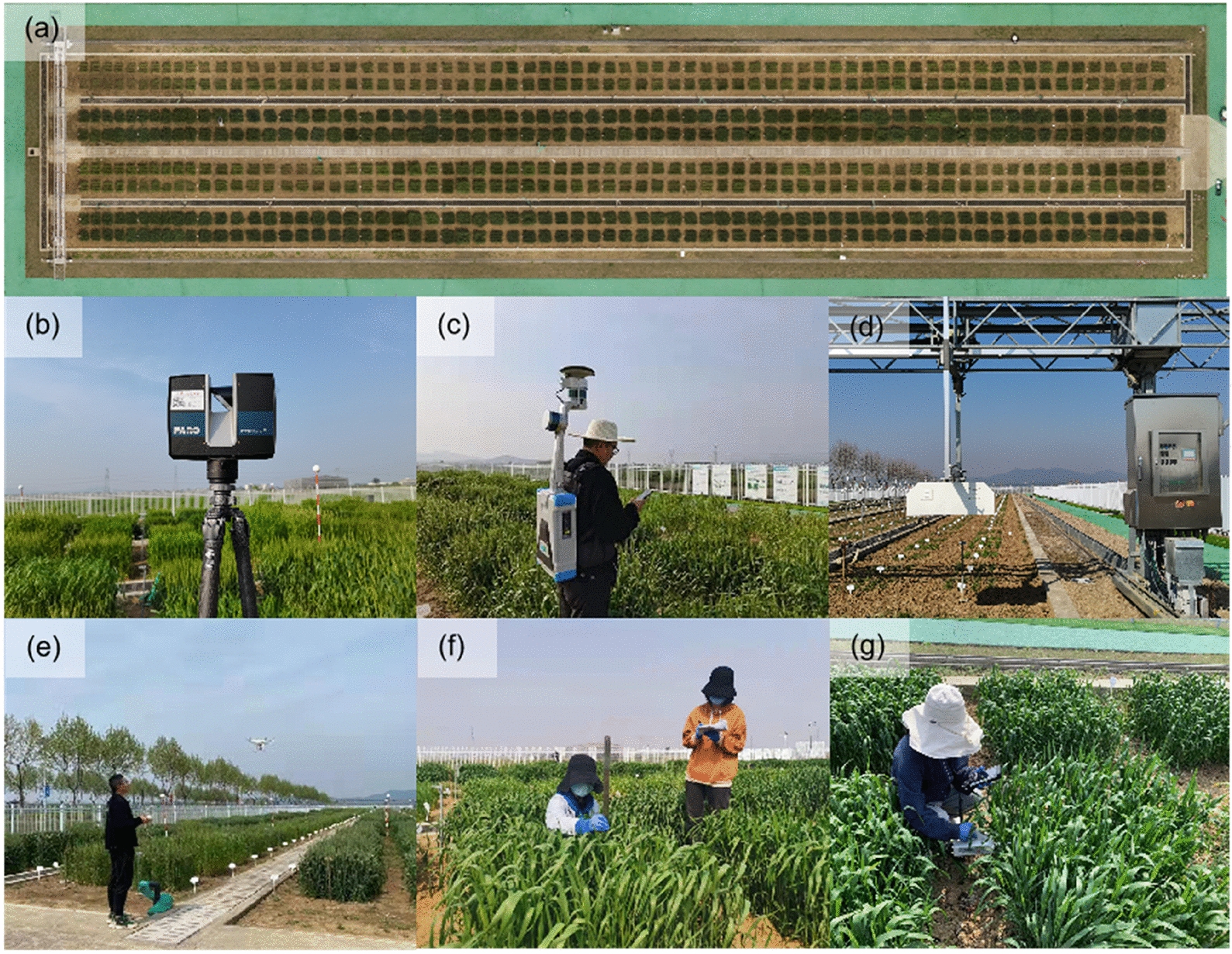
**a** Study area and data collection by **b** terrestrial laser scanning (TLS), **c** backpack laser scanning (BLS), **d** gantry laser scanning (GLS), and **e** digital aerial photogrammetry (DAP); Manual measurement of **f** canopy height (CH) with a ruler and **g** leaf area index (LAI) with the SunScan Canopy Analyzer

The study area was located at the Baima Experimental Station (119°18′71″E, 31°62′00″N) of Nanjing Agricultural University, China. A total of 480 plots were cultivated with 120 wheat varieties, two treatments of nitrogen fertilization (0 and 240 kg/ha), and two replications. The plot size is 1 m × 1 m with a plot spacing of 0.5 m, row spacing of 0.25 m, and sowing density of 300 seeds/m^2^ (Fig. 1a). Different varieties, nitrogen treatments, and growth stages provided diverse canopy structure for further comparison of CH from different data sources.

### Data collection

To make a systematic comparison of different height measurement methods, TLS, BLS, GLS, and DAP were selected to collect 3D data at four key growth stages that were jointing (134 days after seeding/DAS), heading (151 DAS), flowering (174 DAS), and maturity stages (188 DAS). These data were collected around noon (10:00–14:00) on sunny days, when the light and wind conditions are stable and preferred for optical image collection (e.g., DAP), although LiDAR sensors are insensitive to light conditions. Some important technical specifications used by the four 3D sensing systems are presented in Table 1. Meanwhile, field-measured CH and LAI were implemented with a ruler and the Sunscan Canopy Analyzer (*Delta-T Devices Ltd, U.K.*). Finally, different data sources were collected within one day at each growth stage to ensure cross-comparability.

**Table 1:** Technical specifications of TLS, BLS, GLS, and DAP systems

	TLS	BLS	GLS	DAP
System	FARO Focus^3D^ S70	LiBackpack D50	PlantEye F500	DJI Phantom4
Laser wavelength, nm	1550	905	940	RGB image
Field of view, º	H: 360º V: 300º	H: 360º V: − 90º ~ + 90º	~ 53º	94º
Detection range, m	0.60–70 @10% ref	100 @ 20% ref	0.40–1.50	Flight altitude: 10–6000
Data resolution	0.30 mm @ 10 m@ 90% ref	30 mm	H: ~ 0.59 mm V: ~ 1.62 mm	4000 $\times$ 3000 pixels
Weight, kg	4.20	8	8.30	0.138
Size, mm	240 × 200 × 100	960 × 300 × 318	440 × 210 × 99	Wheelbase: 350
Battery capacity, h	4.50	2	unlimited	0.50

#### TLS data

The TLS data was collected using the FARO Focus^3D^ S70 scanner (*FARO Technology Inc, FL, USA*). The sensor weight is 4.2 kg with a size of 240 mm × 200 mm × 100 mm. The field of view is 360° × 300°. The sensor emits lasers at a wavelength of 1550 nm and a pulse emitting rate of 244 kHz. The detection range is 0.6 -70 m with upright incidence to a 10% reflective surface. The scanning accuracy is 0.3 mm @10 m @ 90% reflectance (Table 1).

**Fig. 2:**
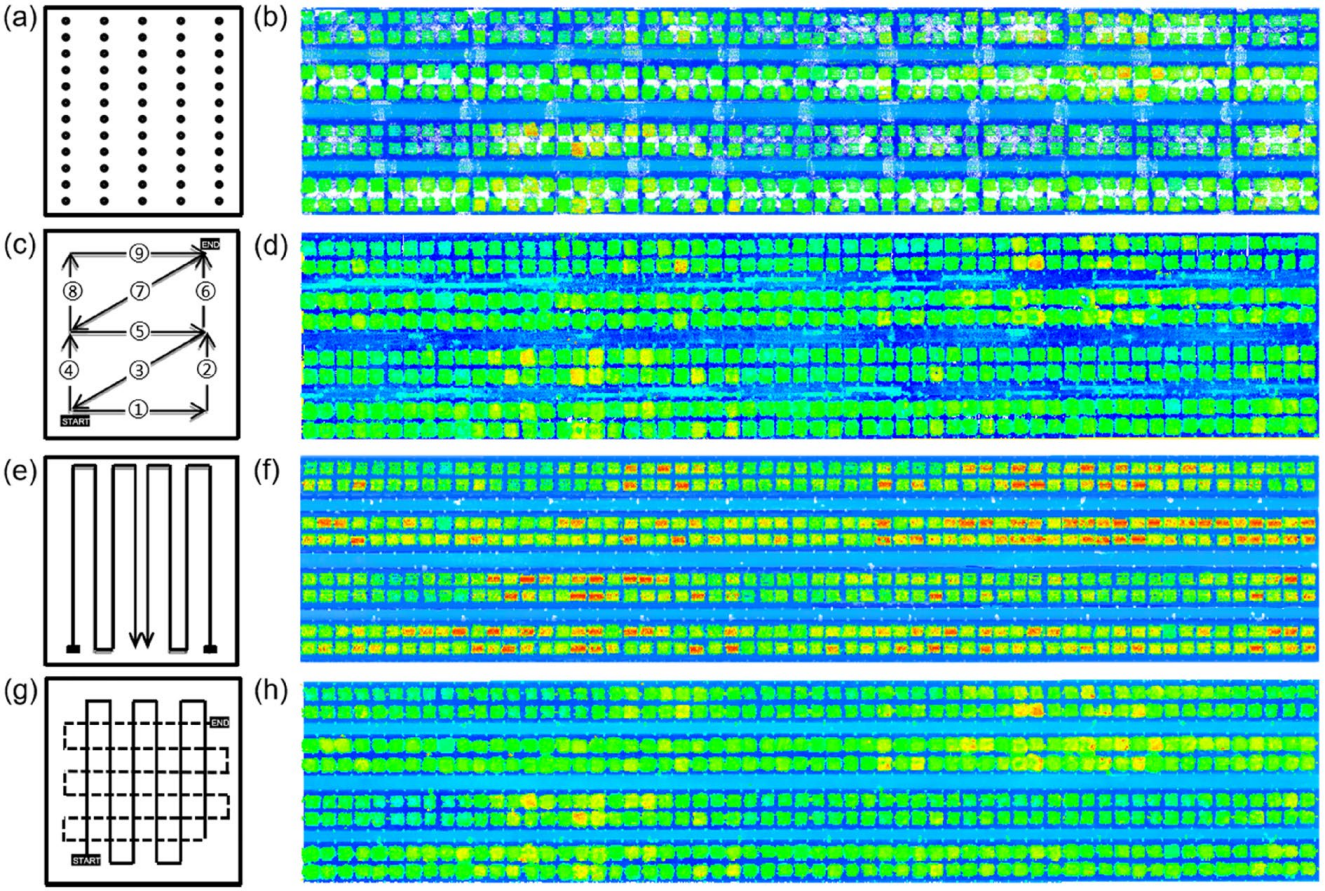
Data acquisition schemes and point clouds collected by different 3D sensing techniques. **a** scanner positions and **b** point cloud of TLS; **c** trajectory and **d** point cloud of BLS; **e** trajectory and **f** point cloud of GLS; and **g** trajectory and **h** point cloud of DAP

The LiDAR sensor was mounted on a tripod (around 1.8 m above the ground) that was placed uniformly in the study area (Fig. 1b). The north–south and east–west distances between the two scanning locations were around 4 m and 7.5 m, respectively. The operating mode of the sensor was set as “*Outdoor within 10 m Scanning Profile*” without color information, which is suitable for acquiring detailed information with high efficiency (~ 5 min/scan) within a short distance (< 10 m) [26]. A total of 65 scans were implemented over the entire wheat field (Fig. 2b), taking around 6 h.

#### BLS data

The BLS data was acquired using the LiBackpack D50 system (*Green Valley International Ltd., Beijing, China*) that was equipped with two Velodyne VLP-16E sensors (*Velodyne Lidar Inc., San Joe, CA, USA*). The system weight is about 8 kg with a size of 960 mm × 300 mm × 318 mm. The field of view is 360° × 180° (− 90º ~  + 90º). The sensor emits lasers at a wavelength of 905 nm and a pulse emitting rate of 30 kHz. The detection range is 100 m with upright incidence to a 20% reflective surface. The scanning accuracy is ± 3 cm (Table 1).

BLS was carried on the shoulder (Fig. 1c), enabling efficient and flexible mobile acquisition. Because BLS uses the SLAM (simultaneous location and mapping) algorithm for data acquisition, the moving trajectory was designed like a series of closed “triangles” (Fig. 2c). The collection time was around 20 min for the whole field.

#### GLS data

The GLS data were acquired by using the FieldScan Phenotyping Platform (Fig. 1c), which is equipped with four high-resolution 3D laser scanners, PlantEye F500 (*Phenospex Inc, Heerlen, The Netherlands*) (Fig. 1d). The sensor weight is around 8.3 kg with a size of 440 mm × 210 mm × 99 mm, and the field of view is around 53°. The sensor emits lasers with a wavelength of 940 nm and a pulse emitting rate of 50 XZ-profiles/s. The ranging distance is between 0.4–1.5 m. The sensors scanning accuracies will decrease with the increase of distance along the vertical height range. The average horizontal and vertical resolutions are around 0.59 mm and 1.62 mm, respectively (Table 1).

The sensor system was carried by a gantry at a height of 1.5 m, and the maximum scan range is 1.1 m. The GLS system traveled automatically in the field with a defined regular trajectory (Fig. 2e). The system repeatedly collected data day and night for the whole field. Each round of collection took around 4.5 h, and then the system slept 1.5 h before the next round of collection. Notably, the integrated software system will remove ground points (i.e., filtering) by setting a height threshold of the lowest 0.28 m in this study, so the maximum detected canopy height of the GLS system is 0.82 m.

#### DAP data

The DAP data was collected using the DJI Phantom4 drone (*SZ DJI Technology Co., Shenzhen, China*) by carrying an RGB camera (Fig. 1e). The camera has a resolution of 4000 pixels × 3000 pixels. The field of view is 94°. Flight missions were planned using the Pix4D Capture software (*PIX4D S.A., Lausanne, Switzerland*). To balance the problem of acquisition accuracy and efficiency [31], we carried out comparisons at different flight altitudes, including 10 m, 20 m, 30 m, and 40 m. The 20 m was selected because its accuracy is comparable to 10 m and higher than 30 m and 40 m (Additional file 1: Fig. S1). Oblique imageries were collected to ensure substantial overlap and reduced systematic errors [23]. Meanwhile, the cross fight was set up, covering an east–west and a north–south flight trajectories, to improve 3D reconstruction accuracy from images (Fig. 2g). Specifically, the forward and side overlaps were both set as 80%. The camera angle during the flight was set to 80° by referring to Rosnell and Honkavaara [51]. Seven ground control points were set up for image quality control in the field. A total of 216 images were collected during a 20 min flight.

#### Field measurements

In this study, the field CH is defined as the vertical distance from the ground to the highest point of a canopy in the natural growth state. In each plot, CHs were measured with a ruler of mm precision at three locations that look uniform and representative. The three replicated measurements were averaged as the reference CH (Fig. 1f) [68]. LAI was defined as half the total intercepting leaf area pre-unit ground area [6]. LAI was measured with a SunScan Canopy Analyzer (*Delta-T Devices Ltd, Cambridge, U.K.*) that has a 1-m light-sensitive probe with 64 equally spaced photodiodes. The SunScan Canopy Analyzer estimates LAI by measuring the gap fraction [49]. In each plot, the probe was inserted into the bottom of the canopy and parallel to the row direction [47, 55]. Three replicated measurements were implemented and averaged as the reference LAI (Fig. 1g).

## Methods

### Data preprocessing

**Fig. 3:**
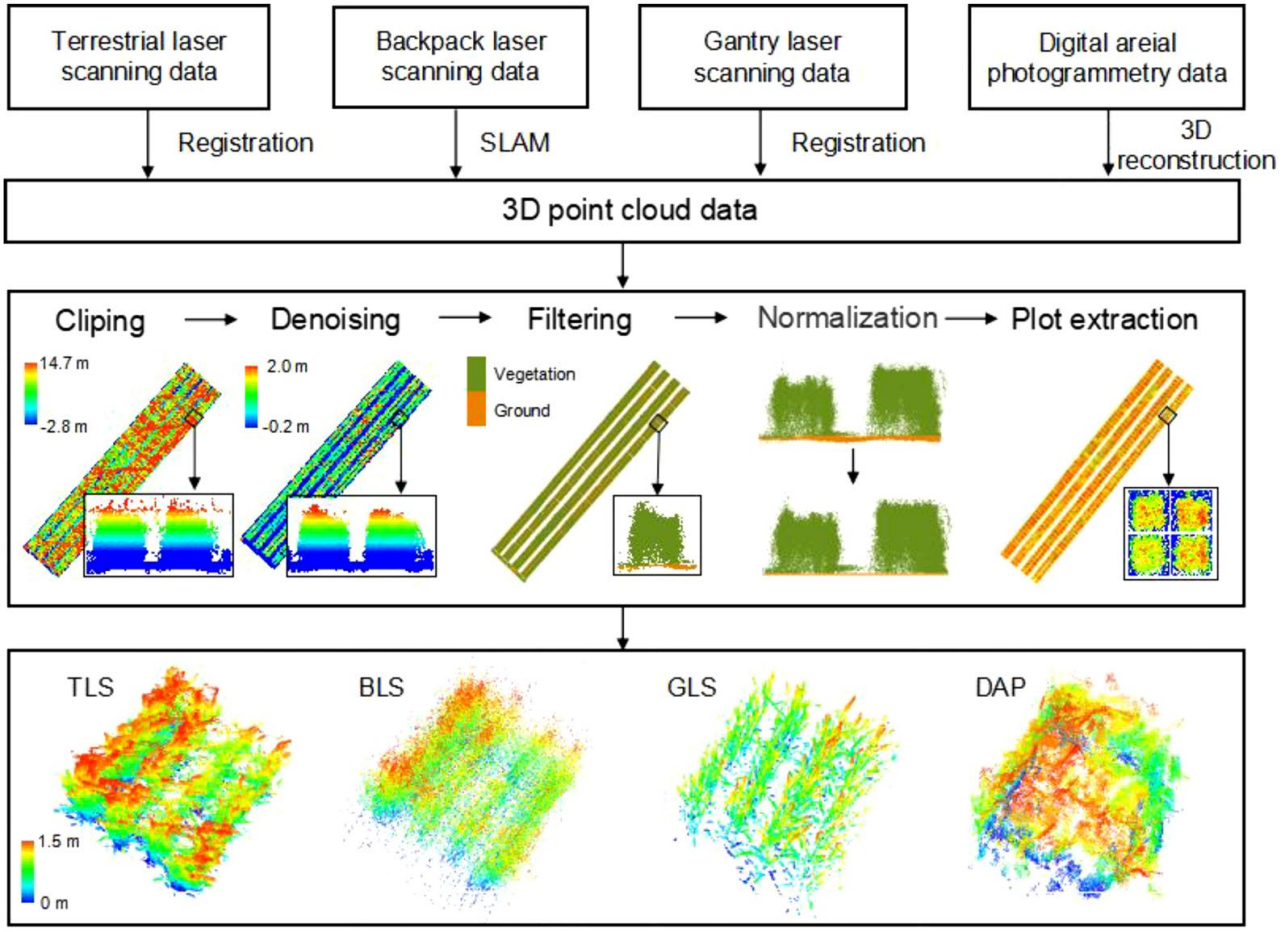
Processing of TLS, BLS, GLS, and DAP data. The processing pipeline was demonstrated using GLS data at the heading stage. SLAM means simultaneous location and mapping

Different 3D sensing data need to be first processed into point clouds with different methods before sharing similar point processing methods. TLS data at different scanning locations were automatically registered to generate a point cloud using SCENE software (*FARO Technology Inc, FL, USA)*. BLS was registered during data collection because the system used the SLAM algorithm [58]. GLS data registration was implemented according to the relative position of sensors and the point features using the commercial HortControl software (*Phenospex Inc, Heerlen, The Netherlands*). DAP images were used to reconstruct the 3D point cloud using the PiX4D mapper software (*Pix4D, Lausanne, Switzerland*). Once the 3D point clouds were generated, the following data processing processes were similar (Fig. 3).

The generated 3D point cloud data were further processed with a standard pipeline using the LiDAR360 software (*Green Valley International Ltd., Beijing, China*), including clipping, denoising, filtering, and normalization (Fig. 3). Clipping and denoising were manually implemented to ensure better accuracy, especially avoiding the loss of points in the sparse DAP and BLS point cloud. Filtering was first implemented using an integrated algorithm (i.e., improved progressive triangulated irregular network densification filtering algorithm), and the automatic results were carefully checked and revised to decrease process errors. Normalization was achieved by subtracting the height of each point from the height of its nearest ground point in the horizontal direction. Specifically, GLS data was filtered with a given height threshold of 0.28 m and normalized during data collection. The normalized 3D point clouds of TLS, BLS, GLS, and DAP were shown in Fig. 2b, d, f, h. Taking pre-processed data at the heading stage as an example, the point density of TLS data is the highest (929,021.12 pts/m^2^), followed by GLS (697,092.18 pts/m^2^), DAP (40,051.30 pts/m^2^), and BLS (17,761.30 pts/m^2^). Meanwhile, the final point resolution, denoted by the average adjacent point distance, from fine to coarse was GLS (1.07 mm), TLS (2.46 mm), DAP (12.73 mm), and BLS (15.02 mm) (Table 2).

**Table 2:** Key information about the data quality of the preprocessed point clouds (taking data at the heading stage as an example) and the roughly estimated platform cost and data cost

Data sources	Point density, pts/m^2^	Point resolution, mm	Data volume, GB	Platform cost, $	Data Cost, h		
					Collection	Preprocessing	Total
TLS	929,021.12	2.46	11.40	46,010.00	5.00	79.20	84.20
BLS	17,761.30	15.02	0.22	70,515.00	0.30	2.30	2.60
GLS	697,092.18	1.07	8.68	1,567,000.00	8.00	2.00	6.50
DAP	40,051.30	12.73	0.48	1253.60	0.50	15.70	16.20

Plot extraction is the prerequisite for CH extraction of each plot. Because different sources of point clouds have their sensor coordinate systems, this study manually aligned these data into the same coordinate origin and north–south directions in LiDAR360 software. After that, 480 plots of different source data at each growth stage can be extracted using a shared plot bounding box map defined manually (Fig. 3).

### Canopy height extraction

CH can be extracted from the normalized point cloud using different statistical metrics. In this study, Hmax, the maximum *z* value of all normalized points, was extracted. Meanwhile, difference height quantiles from 99% quantile height (i.e., H99) to 80% quantile height (i.e., H80) with an interval of 1% were also extracted [27]. These different height representations are compared and the optimal one was selected for comparing different sensing technologies.

### Cross-comparisons of canopy height estimates from field measurement and 3D sensing

The accuracies of the CH measured by different 3D sensing data were compared with the field measurement, and the cross-comparisons of different 3D sensing performances were also evaluated. Specifically, the comparisons between sensor data with field measurement include TLS vs.FM, BLS vs.FM, GLS vs.FM, DAP vs.FM, and the cross-comparisons include TLS vs. BLS, BLS vs. DAP, DAP vs. TLS, TLS vs. GLS, BLS vs. GLS, and DAP vs. GLS.

This study further evaluated the accuracy of different methods with respect to different field-measured CH groups, LAI groups, and GS groups, which are important indicators of canopy structure [41, 42] and affect the accuracy of CH monitoring. Four CH groups were considered, including 0.3–0.6 m (CH1), 0.6–0.8 m (CH2), 0.8–1 m (CH3), and 1–1.4 m (CH4). Each height group contains 360, 918, 501, and 141 plots, respectively. Four LAI groups were separated at 0–2 m^2^/m^2^ (LAI1), 2–4 m^2^/m^2^ (LAI2), 4–6 m^2^/m^2^ (LAI3), and 6–8 m^2^/m^2^ (LAI4). Each group contains 874, 641, 340, and 65 plots, respectively. Four compared growth stages were jointing stages, heading stages, flowering stages, and maturity stages.

Specifically, considering the scanning range and height threshold setting in filtering, the effective maximum height of the GLS system is 0.82 m. Therefore, only the plots that have a maximum measured height lower than 0.82 m were selected for comparison with GLS (1365 plots) in this study. Because there are a few plots belonging to the CH3 group and no plots belonging to the CH4 group, we only evaluated the GLS accuracies of CH1 and CH2 (360 and 918 plots, respectively).

The accuracy between the two compared groups was evaluated by Pearson’s correlation coefficient (*r*), root mean square error (*RMSE*), relative RMSE (*RMSE%*), Bias, and relative Bias (*Bias%*).

1 $$r=\sqrt{1-\frac{\sum {\left({y}_{i}-{\widehat{y}}_{i}\right)}^{2}}{\sum {\left({y}_{i}-\overline{{y}_{i}}\right)}^{2}}}$$

2 $$RMSE=\sqrt{\frac{1}{n}\sum_{i=1}^{n}{({y}_{i}-{\widehat{y}}_{i})}^{2}}$$

3 $$RMSE\%=(\frac{RMSE}{\overline{{y}_{i}}})\times 100$$

4 $$Bias={\sum }_{i=1}^{n}\left({y}_{i}-{\widehat{y}}_{i}\right)/n$$

5 $$Bias\%=(\frac{Bias}{\overline{{y}_{i}}})\times 100$$

where *i* represents a sample index, *n* represents the number of samples, *y*_*i*_ represents reference measurements (e.g., FM), $\widehat{{y}_{i}}$ represents predicted CH from different 3D sensing datasets, and $\overline{{y }_{i}}$ is the mean of *y*_*i*_.

Moreover, the CHs of different data sources were compared in terms of broad-sense heritability (*H*^*2*^). Broad-sense heritability was defined as the proportion of heritability variance, which was computed as the ratio between the genotypic to the total variance [65, 66]. In this study, the interaction effect of different varieties and *N* treatments was considered, i.e., G by E.

6 $${\sigma }_{G}^{2}=\frac{1}{g-1}\sum_{i}{G}_{i}^{2}$$

7 $${\sigma }_{E}^{2}=\frac{1}{e-1}\sum_{j}{E}_{j}^{2}$$

8 $${\sigma }_{GE}^{2}=\frac{1}{(g-1)(e-1)}\sum_{i,j}{GE}_{ij}^{2}$$

9 $${H}^{2}=\frac{{\sigma }_{G}^{2}}{{\sigma }_{G}^{2}+\frac{{\sigma }_{GE}^{2}}{e}+\frac{{\sigma }_{\varepsilon }^{2}}{re}}$$

where ${H}^{2}$ is broad-sense heritability, ${\sigma }_{G}^{2}$, ${\sigma }_{E}^{2}$, and ${\sigma }_{GE}^{2}$ are genotypic variance, environmental variances, and genotype-by-environment interaction variance, respectively. *g* is the number of genotypes, *i* is the index of genotype; *e* is the number of N treatments, *j* is the index of N treatments, and r is the number of replications per genotype.

### Error source analysis

As we know, CHs measured by different methods will not be exactly the same. This study analyzed which data source the error comes from by referring to the method of Wang et al. [71]. First, we calculate the relative residual between the 3D sensing estimated CHs and FM (Eq. 10). Then, screening out the plots where the above calculated relative residuals greater than 20% as the suspicious cases (S) (Eq. 11). The intersections of S_TLS_, S_BLS_, S_GLS_, and S_DAP_ were defined as the errors due to FM (Error_FM) (Eq. 12). Based on Error_FM, the intersection of S_TLS_, S_BLS_, S_GLS_, S_DAP,_ and non-Error_FM was defined as the errors due to TLS (Error_TLS), BLS (Error_BLS), GLS (Error_GLS), and DAP (Error_DAP), respectively (Eq. 13–16). Notably, when regarding TLS or any other 3D sensing datasets as the errors, it is not mean the other three 3D sensing datasets do not contain outliers because the conditions for Error_FM are very strict.

10 $${\Delta }_{\left(a,field\right)}^{i}=\left|{H}_{a}^{i}-{H}_{filed}^{i}\right|/{H}_{field}^{i}$$

11 $${S}_{a}=\left\{{P}^{i}|{\Delta }_{\left(a,field\right)}^{i}\ge 0.2\right\}$$

12 $$Error\_FM=\left\{{P}^{i}|{S}_{TLS}\cap {S}_{BLS}\cap {S}_{GLS}\cap {S}_{DAP}\right\}$$

13 $$Error\_TLS=\left\{{P}^{i}|{S}_{TLS}\cap (!Error\_field)\right\}$$

14 $$Error\_BLS=\left\{{P}^{i}|{S}_{BLS}\cap (!Error\_field)\right\}$$

15 $$Error\_GLS=\left\{{P}^{i}|{S}_{GLS}\cap (!Error\_field)\right\}$$

16 $$Error\_DAP=\left\{{P}^{i}|{S}_{DAP}\cap (!Error\_field)\right\}$$

where* i* is the sample index and ${P}^{i}$ represents sampled data (i.e., a plot). ${\Delta }_{\left(a,field\right)}^{i}$ is the relative residual between 3D sensing-derived CH and FM, ${H}_{a}^{i}$ represent predicted CH, where *a* can be TLS, BLS, GLS, and DAP. Meanwhile, the exclamation mark (!) is the “NOT” in logic operations

## Results

### Canopy height from different 3D sensing datasets

**Fig. 4:**
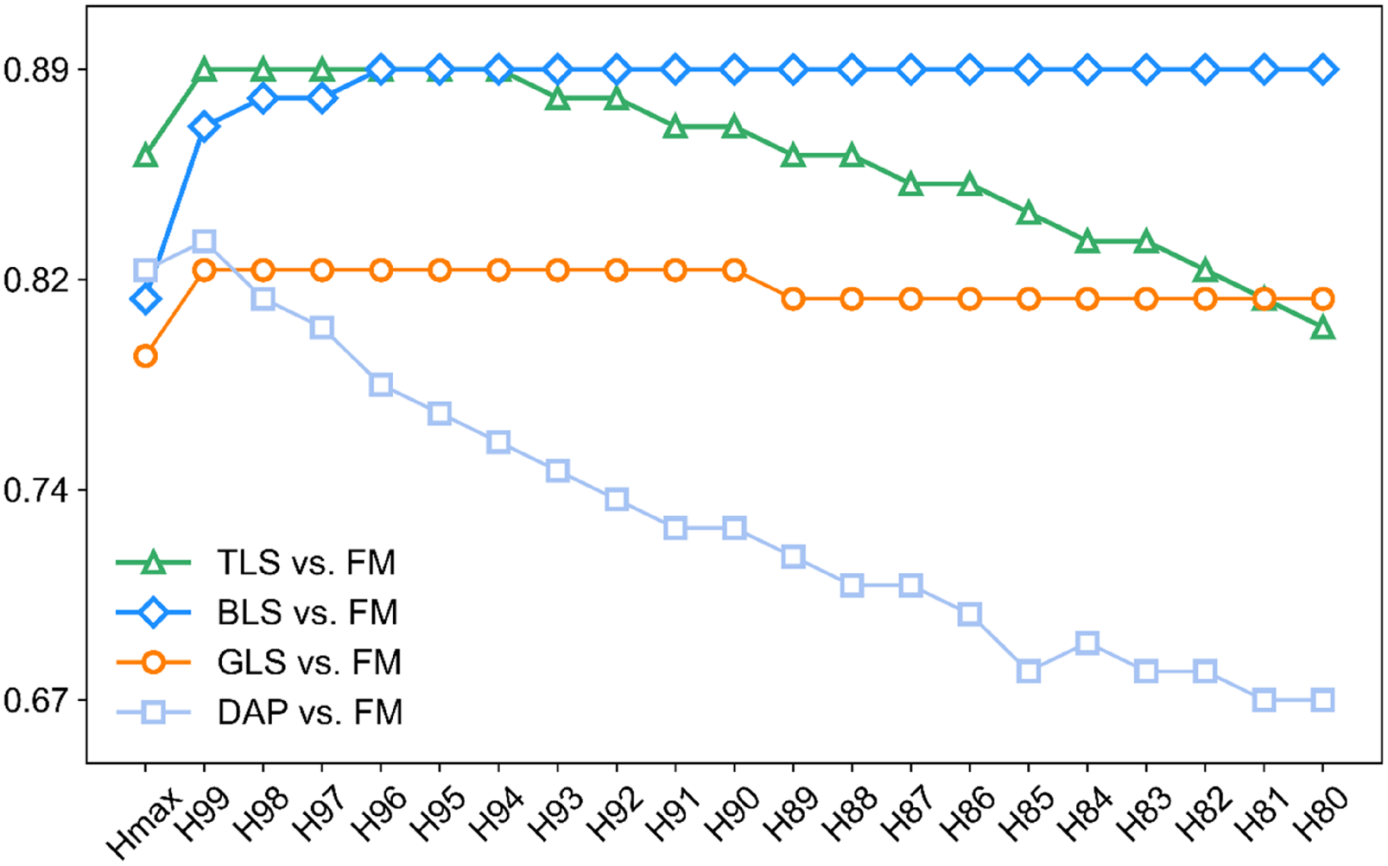
Correlation values between FM and height quantiles (e.g., Hmax and H99) derived from the different 3D sensing data, including TLS, BLS, GLS, and DAP. The green triangle, blue diamond, dark orange circle, and baby blue square represent correlations of TLS vs. FM, BLS vs. FM, GLS vs. FM, and DAP vs. FM, respectively

To fairly compare different 3D sensing datasets for CH estimation, it is important to first explore which height representation metric is optimal according to their correlations with FM. In this study, the influences of different height quantiles for CH extraction were evaluated using point clouds of all stages. The results showed that the evaluation accuracy of TLS, BLS, and GLS were all high and stable when using different height quantiles (Fig. 4). By contrast, height estimation accuracy from DAP data was lower and more sensitive to the selection of height quantiles. According to the highest correlation (Fig. 4) and the lowest error metrics (Additional file 1: Fig. S2), H99 was selected as the best representation of CH for TLS, GLS, and DAP, while H96 was the best for BLS. These best height quantiles (H99 or H96) for each data source was used for all subsequent analysis.

**Fig. 5:**
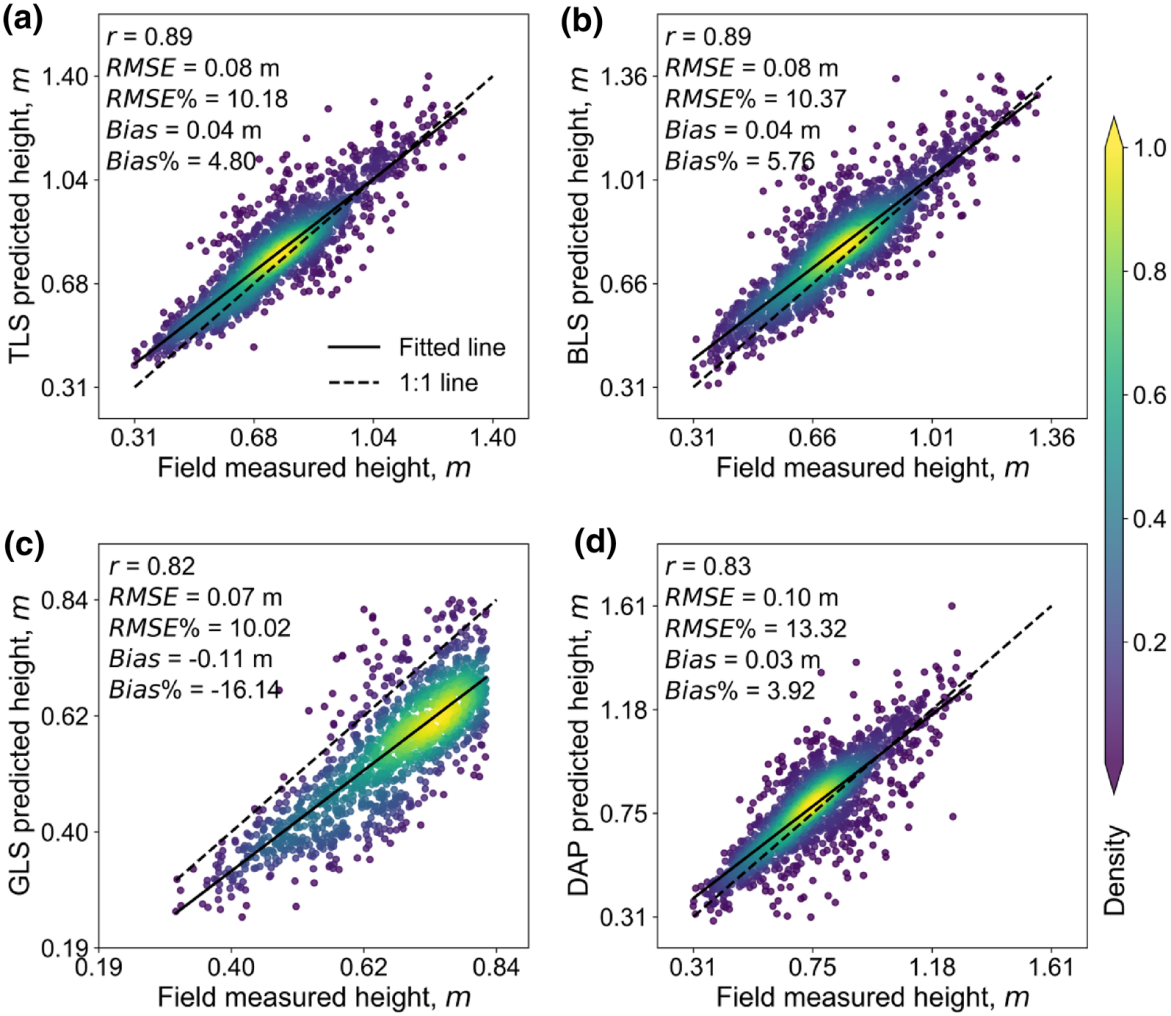
Correlations between FM and predicted heights by different 3D sensing technologies. **a**–**c**, and **d** represent TLS vs. FM, BLS vs. FM, GLS vs.FM, and DAP vs. FM, respectively. The solid line represents the fitted line, and the dashed line represents the 1:1 line. The color bar shows the kernel density value of the point distribution, and the green to yellow represents the increase in kernel density

The best correlations of TLS vs. FM, BLS vs. FM, GLS vs. FM, and DAP vs. FM were 0.89, 0.89, 0.82, and 0.83, respectively (Fig. 5). The fitted lines of TLS, BLS, and DAP were close to the reference lines (1:1) except a little overestimation when CH was small (Fig. 5). In contrast, GLS showed an overall underestimation (Fig. 5c).

**Fig. 6:**
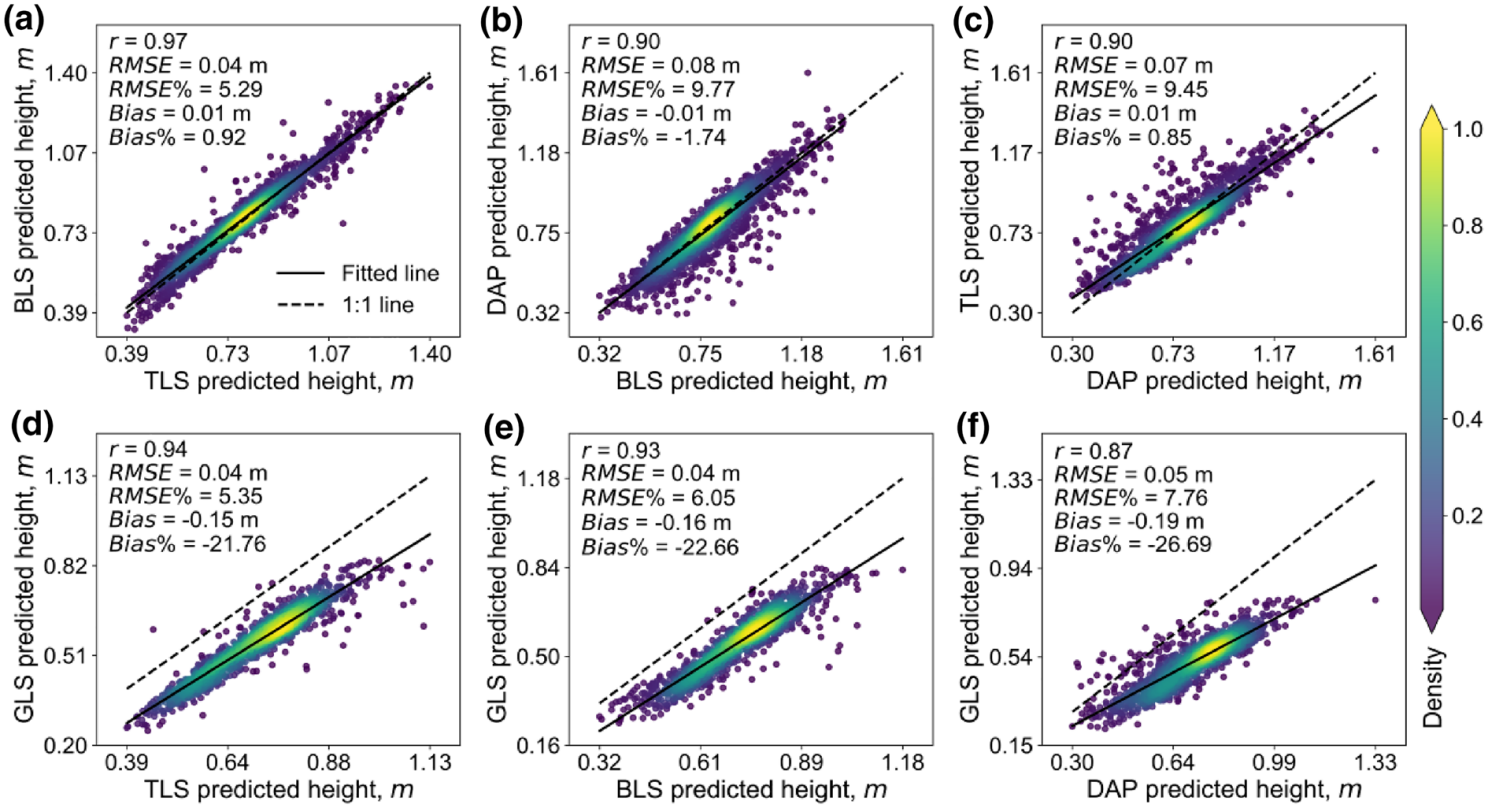
Canopy height correlations between different 3D sensing estimates. **a**–**e**, and **f** represent TLS vs. BLS, BLS vs. DAP, DAP vs. TLS, TLS vs. GLS, BLS vs. GLS, and DAP vs. GLS, respectively. The solid line represents the fitted line, and the dashed line represents the 1:1 line. The color bar shows the kernel density value of the point distribution, and the green to yellow represents the increase in kernel density

Cross-comparisons among different sensor datasets showed higher correlations (*r*) ranging from 0.87 to 0.97, which was much higher than the above comparisons with FM (0.82–0.89). The highest correlation value is 0.97 between TLS and BLS (Fig. 6a), followed by TLS vs. GLS (r = 0.94) (Fig. 6d), BLS vs. GLS (r = 0.93) (Fig. 6e), DAP vs. TLS (r = 0.90) (Fig. 6c), BLS vs. DAP (r = 0.90) (Fig. 6b), and DAP vs. GLS (r = 0.87) (Fig. 6f). Among them, DAP had a relative lager RMSE with other sensing datasets (RMSE > 0.05 m, Fig. 6b, c, f), especially the comparison with BLS (RMSE = 0.08 m, Fig. 6b). Moreover, the fitting *Bias* are all very small (0.01 m) except for comparisons with GLS (Fig. 6d, e, f). Although GLS showed an overall underestimation, it still keeps a low RMSE (0.04 m-0.05 m) with other 3D sensing datasets.

### Comparing canopy height measurement of different methods among different canopy height groups

The correlation coefficients of CHs derived from 3D sensing and FM decreased obviously when evaluated with respect to different subgroups of CH (*r* < 0.71). Similarly, the correlation coefficients of cross-comparisons of different 3D sensing also decreased, although the largest *r* was up to 0.93 (Table 3).

**Table 3:** Detailed statistics on comparing canopy height measurement methods

CH group	RMSE, m (RMSE%)	Bias, m (Bias%)	*r*	RMSE, m (RMSE%)	Bias, m (Bias%)	*r*
	TLS vs. FM			BLS vs. FM		
CH1	0.06 (10.92)	0.05 (10.24)	0.66	0.07 (13.65)	0.06 (12.60)	0.65
CH2	0.07 (10.48)	0.05 (6.79)	0.56	0.08 (10.96)	0.06 (7.85)	0.54
CH3	0.08 (9.57)	0.01 (0.95)	0.49	0.08 (8.75)	0.01 (1.48)	0.52
CH4	0.09 (7.98)	0.01 (0.86)	0.59	0.08 (7.58)	0.01 (0.99)	0.64
Mean	0.08 (9.74)	0.03 (4.71)	0.58	0.08 (10.23)	0.04 (5.73)	0.59
	GLS vs. FM			DAP vs. FM		
CH1	0.06 (11.39)	− 0.09 (− 17.96)	0.64	0.07 (12.91)	0.04 (8.15)	0.71
CH2	0.07 (9.82)	− 0.11 (− 15.49)	0.56	0.09 (13.10)	0.05 (6.76)	0.51
CH3	–	–	–	0.11 (12.79)	0.00 (− 0.17)	0.36
CH4	–	–	–	0.14 (12.25)	0.02 (− 1.49)	0.50
Mean	0.06 (10.6)	− 0.10 (− 16.72)	0.60	0.10 (12.76)	0.02 (3.31)	0.52
	TLS vs. BLS			TLS vs. GLS		
CH1	0.05 (8.89)	0.01 (2.15)	0.84	0.03 (5.32)	0.14 (− 25.57)	0.92
CH2	0.04 (4.98)	0.01 (0.99)	0.91	0.04 (5.21)	0.16 (− 20.86)	0.88
CH3	0.04 (4.30)	0.00 (0.52)	0.91	–	–	–
CH4	0.04 (3.71)	0.00 (0.13)	0.93	–	–	–
Mean	0.04 (5.47)	0.01 (0.95)	0.89	0.03 (5.27)	− 0.15 (− 23.22)	0.90
	BLS vs. DAP			BLS vs. GLS		
CH1	0.06 (10.80)	− 0.02 (− 3.95)	0.75	0.04 (7.47)	0.16 (− 27.14)	0.82
CH2	0.07 (8.98)	− 0.01 (− 1.00)	0.77	0.04 (5.31)	0.17 (− 21.64)	0.88
CH3	0.09 (9.80)	− 0.01 (− 1.62)	0.69	–	–	-
CH4	0.11 (9.46)	− 0.03 (− 2.45)	0.74	–	–	-
Mean	0.08 (9.76)	− 0.02 (− 2.26)	0.74	0.04 (6.39)	− 0.16 (− 24.39)	0.85
	DAP vs. TLS			DAP vs. GLS		
CH1	0.04 (7.58)	0.01 (1.93)	0.83	0.06 (13.67)	0.13 (31.82)	0.79
CH2	0.06 (7.83)	0.00 (0.03)	0.75	0.07 (12.38)	0.16 (26.33)	0.73
CH3	0.07 (8.36)	0.01 (0.01)	0.65	–	–	–
CH4	0.07 (6.66)	0.03 (0.03)	0.75	–	–	–
Mean	0.06 (7.61)	0.01 (1.37)	0.74	0.05 (7.95)	− 0.15 (− 22.49)	0.76

The top side of the table showed the evaluation results of 3D sensing datasets with FM; the bottom side of the table showed the results of 3D sensing datasets cross-comparisons. RMSE and RMSE%, Bias and Bias%, and correlation coefficient (r) were given for distinct canopy height (CH) groups. The underlined values were the best result for each CH group among different comparisons

- represents the comparison was not available due to the limited ranging ability of the GLS system

**Fig. 7:**
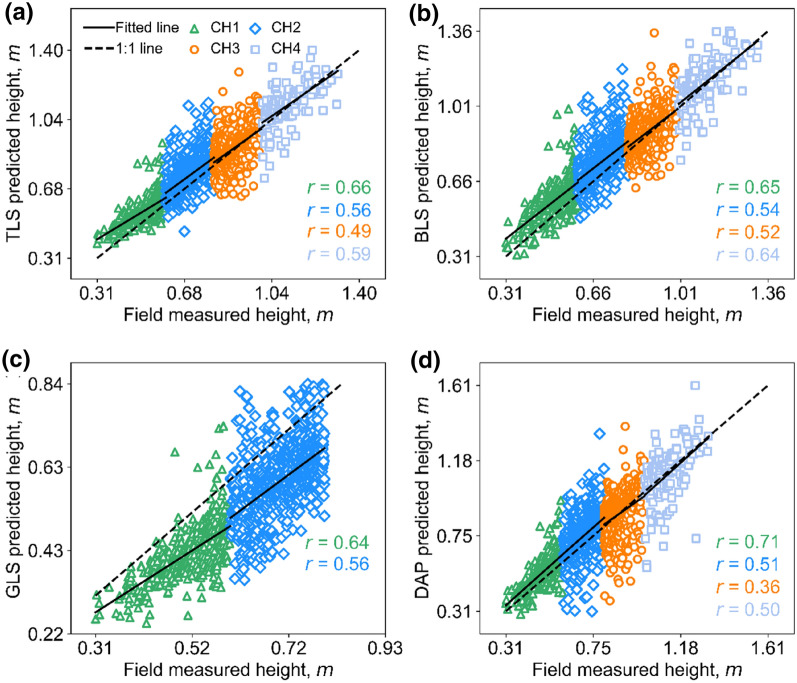
Correlations between FM heights and predicted heights by different 3D sensing technologies under four canopy height (CH) groups. **a**–**d** represent TLS vs. FM, BLS vs. FM, GLS vs. FM, and DAP vs. FM, respectively. The green triangle, blue diamond, dark orange circle, and baby blue square represent the CH1, CH2, CH3, and CH4 groups, respectively. The solid line represents the fitted line, and the dashed line represents the 1:1 line

As for comparing 3D sensing with FM, GLS was the best according to the highest mean *r* (0.60), followed by BLS (mean *r* = 0.59), TLS (mean* r* = 0.58), and DAP (mean *r* = 0.52) (Table 3). From the prospect of subgroup comparisons, the best methods for estimating CH1, CH2, CH3, and CH4 were DAP (mean *r* = 0.71), TLS (mean *r* = 0.56), BLS (mean *r* = 0.52), and BLS (mean *r* = 0.64), respectively (Table 3). The fitting lines of TLS, BLS, and DAP were very close to the reference lines in CH3 and CH4 groups, while slight overestimation appeared in CH1 and CH2 groups (Fig. 7). Consistently, GLS showed underestimation in both CH1 and CH2 groups (Fig. 7c).

The cross-comparisons of different 3D methods showed much higher correlation values. Among them, TLS vs. GLS showed the highest correlation (mean *r* = 0.90), followed by TLS vs. BLS (mean* r* = 0.89), BLS vs. GLS (mean *r* = 0.85), DAP vs. GLS (mean *r* = 0.76), DAP vs. TLS (mean *r* = 0.74), and BLS vs. DAP (mean *r* = 0.74). From the perspective of subgroup comparisons, the most consistent method for estimating CH1 was TLS vs. GLS, and the most consistent methods for estimating CH2, CH3, and CH4 were always TLS vs. BLS (Table 3).

The fitted lines for TLS vs. BLS and BLS vs. DAP were both close to 1:1 for different CH groups (Additional file 1: Fig. S3 a, b). DAP vs. TLS showed overestimation at low heights and underestimation at high heights for each height group (Additional file 1: Fig. S3 c). Underestimations also almost existed in comparisons between GLS and other 3D sensing datasets at every CH group (Additional file 1: Fig. S3 d-f). The fitted line of GLS vs. TLS was nearly parallel to the reference line, while underestimations to other 3D data become more obvious with height growth.

### Comparing canopy height measurement of different methods among different LAI groups

The correlation coefficients of CHs derived from 3D sensing and FM only decreased slightly (mean *r* = 0.79 to 0.87) with respect to different LAI groups. Likewise, the correlation coefficients of cross-comparisons of different 3D sensing also decreased slightly (mean *r* = 0.84 to 0.96), with little change for TLS vs. BLS (Table 4).

**Table 4:** Detailed statistics on comparing canopy height measurement methods

LAI group	RMSE, m (RMSE%)	Bias, m (Bias%)	*r*	RMSE, m (RMSE%)	Bias, m (Bias%)	*r*
	TLS vs. FM			BLS vs. FM		
LAI1	0.07 (9.11)	0.03 (4.71)	0.93	0.06 (8.78)	0.04 (5.13)	0.93
LAI2	0.09 (11.43)	0.04 (5.32)	0.86	0.09 (11.74)	0.05 (7.11)	0.85
LAI3	0.07 (9.79)	0.03 (3.88)	0.83	0.08 (10.64)	0.03 (4.68)	0.79
LAI4	0.07 (8.98)	0.04 (5.46)	0.86	0.07 (9.10)	0.05 (6.16)	0.84
Mean	0.07 (9.83)	0.04 (4.84)	0.87	0.07 10.06)	0.04 (5.77)	0.85
	GLS vs. FM			DAP vs. FM		
LAI1	0.05 (7.66)	− 0.09 (− 14.98)	0.91	0.08 (11.51)	0.02 (2.43)	0.89
LAI2	0.08 (12.05)	− 0.10 (− 14.99)	0.73	0.13 (16.53)	0.04 (5.61)	0.77
LAI3	0.06 (8.86)	− 0.14 (− 19.58)	0.78	0.07 (9.89)	0.03 (4.09)	0.77
LAI4	0.06 (7.85)	− 0.13 (− 18.74)	0.73	0.06 (8.55)	0.04 (5.78)	0.82
Mean	0.06 (9.10)	− 0.12 (− 17.07)	0.79	0.09 (11.62)	0.03 (4.48)	0.81
	TLS vs. BLS			TLS vs. GLS		
LAI1	0.04 (5.05)	0.00 (0.40)	0.97	0.03 (4.46)	− 0.15 (− 21.93)	0.97
LAI2	0.04 (5.25)	0.01 (1.70)	0.97	0.04 (5.40)	− 0.15 (− 20.89)	0.94
LAI3	0.04 (5.68)	0.01 (0.77)	0.94	0.04 (6.02)	− 0.16 (− 22.76)	0.90
LAI4	0.04 (4.74)	0.01 (0.67)	0.95	0.05 ( 6.33)	− 0.17 (− 22.32)	0.82
Mean	0.04 (5.18)	0.01 (0.88)	0.96	0.04 (5.55)	− 0.16 (− 21.98)	0.91
	BLS vs. DAP			BLS vs. GLS		
LAI1	0.07 (8.64)	0.02 (− 2.56)	0.93	0.04 (5.48)	− 0.15 (− 22.54)	0.95
LAI2	0.09 (11.08)	0.01 (− 1.40)	0.89	0.04 (5.51)	− 0.17 (− 22.21)	0.94
LAI3	0.06 (8.52)	0.00 (− 0.56)	0.82	0.05 (7.00)	− 0.17 (− 23.52)	0.86
CH4	0.07 (8.33)	0.00 (− 0.36)	0.80	0.06 (7.38)	− 0.18 (− 23.24)	0.74
Mean	0.07 (9.14)	− 0.01 (− 1.22)	0.86	0.05 (6.34)	− 0.17 (− 22.88)	0.87
	DAP vs. TLS			DAP vs. GLS		
LAI1	0.06 (8.21)	0.02 (− 2.18)	0.94	0.04 (6.48)	− 0.13 (− 20.08)	0.93
LAI2	0.10 (12.06)	0.00 (0.28)	0.87	0.07 (9.79)	− 0.15 (− 21.10)	0.80
LAI3	0.06 (8.14)	0.00 (0.20)	0.84	0.06 (7.96)	− 0.17 (− 23.75)	0.81
LAI4	0.06 (8.12)	0.00 (0.31)	0.82	0.05 (6.33)	− 0.18 (− 23.65)	0.81
Mean	0.07 (9.32)	0.00 (0.36)	0.87	0.06 (7.64)	− 0.16 (− 22.14)	0.84

The top side of the table showed the evaluation results of 3D sensing datasets with FM; the bottom side of the table showed the results of 3D sensing datasets cross-comparisons. RMSE and RMSE%, Bias and Bias%, and correlation coefficient (r) were given for distinct leaf area index (LAI) groups. The underlined values were the best result for each LAI group among different comparisons

**Fig. 8:**
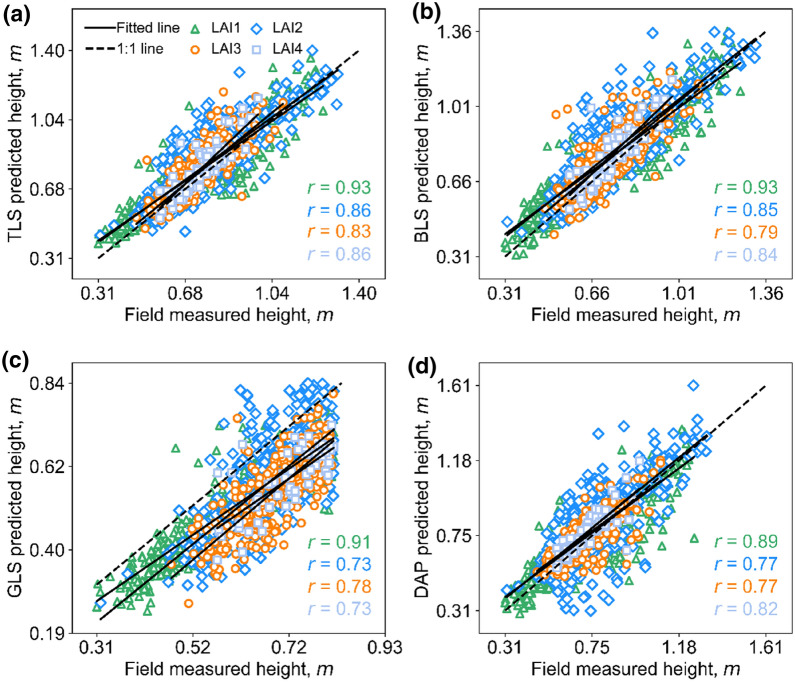
Correlations between FM heights and predicted heights by different 3D sensing technologies under four leaf area index (LAI) groups. **a**–**d** represent TLS vs. FM, BLS vs. FM, GLS vs.FM, and DAP vs. FM, respectively. The green triangle, blue diamond, dark orange circle, and baby blue square represent the LAI1, LAI2, LAI3, and LAI4 groups, respectively. The solid line represents the fitted line, and the dashed line represents the 1:1 line

As for comparing 3D sensing with FM, TLS was the best according to the highest mean *r* (0.87), followed by BLS (mean *r* = 0.85), DAP (mean *r* = 0.81), and GLS (mean *r* = 0.79). From the presence of subgroup comparisons, the best method for estimating the height of the LAI1 group were BLS (mean *r* = 0.93) and TLS (mean *r* = 0.93), while the best method for LAI2, LAI3, and LAI4 was always TLS (mean *r* > 0.83) (Table 4). The fitting lines of TLS, BLS, and DAP were very close to the reference lines in all LAI groups, while GLS showed underestimation in all LAI groups (Fig. 8).

As for cross-comparison of different 3D methods, TLS vs. BLS showed the highest correlation (mean *r* = 0.96), followed by TLS vs. GLS (mean *r* = 0.91), BLS vs. GLS (mean *r* = 0.87), DAP vs. TLS (mean *r* = 0.87), BLS vs. DAP (mean *r* = 0.86), and DAP vs. GLS (mean *r* = 0.84). From the perspective of subgroup comparisons, the most consistent methods for estimating LAI1 were TLS vs. BLS and TLS vs. GLS (mean *r* = 0.97). Besides, the most consistent methods for estimating LAI3 and LAI4 were still TLS vs. BLS (mean *r* = 0.94 and 0.95) (Table 4).

The fitted line for TLS vs. BLS almost coincided with the reference line (Additional file 1: Fig. S4 a). The fitted lines for BLS vs. DAP and DAP vs. TLS were also relatively close to the reference line, but they became worse when LAI increased (Additional file 1: Fig. S4b, c). Underestimations also existed in comparisons between GLS and other 3D sensing datasets at all LAI groups, and the correlations decreased when LAI increased (Additional file 1: Fig. S4d-f).

### Comparing canopy height measurement of different methods among different GS groups

The correlation coefficients of CHs derived from 3D sensing and FM were less accurate (mean r = 0.65 to 0.83) with regard to different GS groups, especially for GLS vs. FM. By contrast, the correlation coefficients of cross-comparisons of different 3D sensing data decreased slightly (mean *r* = 0.80 to 0.94) (Table 5).

**Table 5:** Detailed statistics on comparing height measurement methods

GS group	RMSE, m (RMSE%)	Bias, m (Bias%)	*r*	RMSE, m (RMSE%)	Bias, m (Bias%)	*r*
	TLS vs. FM			BLS vs. FM		
J	0.04 (6.69)	0.02 (3.37)	0.88	0.05 (9.58)	0.03 (5.32)	0.81
H	0.05 (6.64)	0.04 (5.42)	0.92	0.05 (6.49)	0.04 (4.89)	0.91
F	0.10 (11.82)	0.03 (4.10)	0.76	0.10 (12.03)	0.05 (6.41)	0.76
M	0.08 (9.85)	0.05 (5.99)	0.76	0.07 (9.16)	0.05 (6.26)	0.75
Mean	0.07 (8.75)	0.04 (4.72)	0.83	0.07 (9.32)	0.04 (5.72)	0.81
	GLS vs. FM			DAP vs. FM		
J	0.05 (8.96)	− 0.13 (− 22.39)	0.79	0.05 (8.94)	0.03 (5.25)	0.89
H	0.05 (7.00)	− 0.10 (− 13.84)	0.72	0.05 (6.56)	0.05 (5.75)	0.92
F	0.08 (10.96)	− 0.08 (− 10.99)	0.45	0.14 (16.58)	0.03 (4.13)	0.64
M	0.06 (7.94)	− 0.11 (− 14.74)	0.62	0.11 (14.18)	0.01 (0.85)	0.60
Mean	0.06 (8.71)	− 0.10 (− 15.49)	0.65	0.09 (11.57)	0.03 (3.99)	0.76
	TLS vs. BLS			TLS vs. GLS		
J	0.04 (7.61)	0.01 (1.89)	0.87	0.03 (5.15)	− 0.15 (− 24.93)	0.93
H	0.03 (3.33)	0.00 (− 0.50)	0.98	0.03 (3.88)	− 0.15 (− 20.19)	0.91
F	0.04 (4.44)	0.02 (2.22)	0.97	0.03 (4.28)	− 0.17 (− 20.79)	0.92
M	0.04 (5.26)	0.00 (0.25)	0.92	0.05 (6.07)	− 0.16 (− 20.44)	0.76
Mean	0.04 (5.16)	0.01 (0.97)	0.94	0.04 (4.84)	− 0.16 (− 21.59)	0.88
	BLS vs. DAP			BLS vs. GLS		
J	0.06 (9.68)	0.00 (− 0.07)	0.85	0.04 (6.58)	− 0.16 (− 26.33)	0.88
H	0.05 (5.81)	0.01 (0.82)	0.93	0.04 (4.78)	− 0.15 (− 20.42)	0.86
F	0.08 (9.24)	− 0.02 (− 2.15)	0.89	0.03 (3.49)	− 0.18 (− 22.29)	0.95
M	0.10 (11.93)	− 0.04 (− 5.09)	0.70	0.05 ( 6.65)	− 0.16 (− 20.92)	0.70
Mean	0.07 (9.17)	− 0.01 (− 1.62)	0.84	0.04 (5.51)	− 0.16 (− 22.52)	0.86
	DAP vs. TLS			DAP vs. GLS		
J	0.03 (5.10)	− 0.01 (− 1.78)	0.92	0.04 (7.05)	− 0.16 (− 26.27)	0.86
H	0.04 (5.27)	0.00 (-0.31)	0.95	0.03 (4.54)	− 0.15 (− 20.41)	0.87
F	0.07 (8.52)	0.00 (− 0.02)	0.87	0.05 (5.87)	− 0.17 (− 21.14)	0.84
M	0.09 (10.98)	0.04 (5.10)	0.68	0.06 (7.76)	− 0.13 (− 17.09)	0.61
Mean	0.06 (7.47)	0.01 (0.74)	0.86	0.05 (6.30)	− 0.15 (− 21.23)	0.80

The top side of the table showed the evaluation results of 3D sensing datasets with FM; the bottom side of the table showed the results of 3D sensing datasets cross-comparisons. RMSE and RMSE%, Bias and Bias%, and correlation coefficient (r) were given for distinct growth stage (GS) groups. The underlined values were the best result for each GS group among different comparisons

J, H, F, and M represent jointing, heading, flowering, and maturity stages

**Fig. 9:**
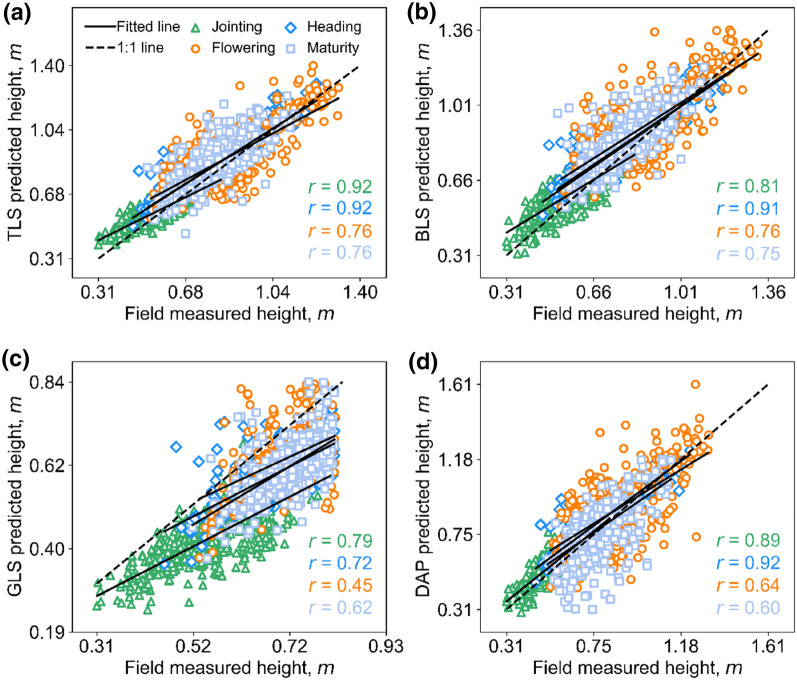
Correlations between FM heights and predicted heights by different 3D sensing technologies under four growth stages (GS) groups. **a**–**d** represent TLS vs. FM, BLS vs. FM, GLS vs.FM, and DAP vs. FM, respectively. The green triangle, blue diamond, dark orange circle, and baby blue square represent the jointing stage, heading stage, flowering stage, and maturity stage respectively. The solid line represents the fitted line, and the dashed line represents the 1:1 line

As for comparing 3D sensing with FM, TLS was the best according to the highest mean *r* (0.83), followed by BLS (mean *r* = 0.81), DAP (mean *r* = 0.76), and GLS (mean *r* = 0.65) (Table 5). From the perspective of subgroup comparisons, DAP was the best method for estimating CH at the jointing stage (mean *r* = 0.89). Moreover, TLS was also the best method for the heading, flowering, and maturity stages (Table 5). The fitting lines of TLS, BLS, and DAP were very close to the reference lines, especially at the heading stage (*r* = 0.72–0.92). However, GLS showed underestimation at all growth stages, which was more obvious at late stages (Fig. 9c).

As for cross-comparison of different 3D methods, TLS vs. BLS showed the highest correlation (mean *r* = 0.94), followed by TLS vs. GLS (mean *r* = 0.88), BLS vs. GLS (mean *r* = 0.86), DAP vs. TLS (mean *r* = 0.86), BLS vs. DAP (mean *r* = 0.84), and DAP vs. GLS (mean *r* = 0.80). From the perspective of subgroup comparisons, the most consistent method for estimating the jointing stage was TLS vs. GLS (mean *r* = 0.93), while the best methods for heading, flowering, and maturity stages were TLS vs. BLS (mean *r* = 0.92–0.98) (Table 5).

The fitted lines of TLS vs. BLS and BLS vs. DAP were closer to the reference line than DAP vs. TLS (Additional file 1: Fig. S5 a, c, e). Underestimations also existed in comparisons between GLS and other 3D sensing datasets at every GS group, especially at the maturity stage (Additional file 1: Fig. S5 d-f).

### Comparing the broad sense heritability of canopy height measurement from different methods

This study found the *H*^*2*^ of CH derived from 3D sensing datasets was overall higher than FM no matter analyzed with CH, LAI, or GS groups (Table 6). At different CH groups, TLS showed the highest *H*^*2*^ (mean* H*^*2*^ = 0.73), followed by BLS (mean *H*^*2*^ = 0.70), GLS (mean *H*^*2*^ = 0.66), DAP (mean *H*^*2*^ = 0.66), and FM (mean *H*^*2*^ = 0.60). The *H*^*2*^ of the lower CH group (CH1) derived from 3D sensing was much larger than the higher CH group (CH2). At different LAI groups, TLS also showed the highest *H*^*2*^ (mean *H*^*2*^ = 0.90), followed by GLS (mean *H*^*2*^ = 0.86), BLS (mean *H*^*2*^ = 0.85), DAP (mean *H*^*2*^ = 0.84), and FM (mean *H*^*2*^ = 0.83). At different GS groups, TLS also showed the highest *H*^*2*^ (mean* H*^*2*^ = 0.89), followed by BLS (mean *H*^*2*^ = 0.85), GLS (mean *H*^*2*^ = 0.81), DAP (mean *H*^*2*^ = 0.79), and FM (mean *H*^*2*^ = 0.77). Overall, *H*^*2*^ of LiDAR-derived CH was larger than that derived from DAP, and *H*^*2*^ of all 3D sensing-derived CH was larger than FM. The overall heritability in the later growth period decreased, especially in the maturity stage.

**Table 6:** The values of Broad-sense heritability (*H*^*2*^) from different 3D sensing datasets with regard to different canopy height (CH), leaf area index (LAI), and growth stage (GS) groups

	FM	TLS	BLS	GLS	DAP
CH group					
CH1	0.58	0.81	0.77	0.73	0.74
CH2	0.61	0.65	0.64	0.59	0.57
CH3	–	–	–	–	–
CH4	–	–	–	–	–
Mean	0.60	**0.73**	**0.70**	**0.66**	**0.66**
LAI group					
LAI1	0.83	0.90	0.85	0.86	0.84
LAI2	–	–	–	–	–
LAI3	–	–	–	–	–
LAI3	–	–	–	–	–
Mean	0.83	**0.90**	**0.85**	**0.86**	**0.84**
GS group					
Jointing	0.79	0.90	0.85	0.86	0.83
Heading	0.83	0.90	0.85	0.86	0.84
Flowering	0.77	0.94	0.86	0.79	0.85
Maturity	0.70	0.82	0.85	0.73	0.62
Mean	0.77	**0.89**	**0.85**	**0.81**	**0.79**

The calculation of H^2^ of a variety was based on the variety’s four plot CHs, including two N treatments and two replicates. Because only CHs under 0.82 m of FM were used for comparison, the H^2^ value of varieties can be calculated only when all four plots’ CH of a variety were below 0.82 m. - represents no varieties within the group that meet the above conditions. The underlined values represent the best results among different height measurement methods (each row) with regard to different subgroups of CH, LAI, and GS. The bold values were the mean vaules of different 3D sensing datasets (each column) across all subgroups of CH, LAI, and GS

## Discussions

### Height quantities of 3D point cloud affect the best estimates of canopy height

Height quantities have been widely used for depicting CH due to their insensitivity to noisy points [19]. However, it has been found that different height quantiles may be suitable for different 3D data with regard to different crop types [38] and sensor types [43].

In this study, we explored the effects of height quantities on the accuracy of height estimation from four kinds of 3D sensing techniques by collecting 1920 wheat plots of various varieties and nitrogen treatments at four growth stages. Our results found that H99 was the best CH quantile of TLS, GLS, and DAP, while H96 was the best for BLS data (Fig. 4). These results are reasonable considering previous studies found the best height quantiles mainly located between H90 and H99, especially near H99, such as the best height quantile for maize was H99 (R^2^ = 0.9) [46] and H99.9 [39], for wheat was H99.5 (R^2^ = 0.90) [43], and for soybean was H99.9 (R^2^ > 0.85) [38].

**Fig. 10:**
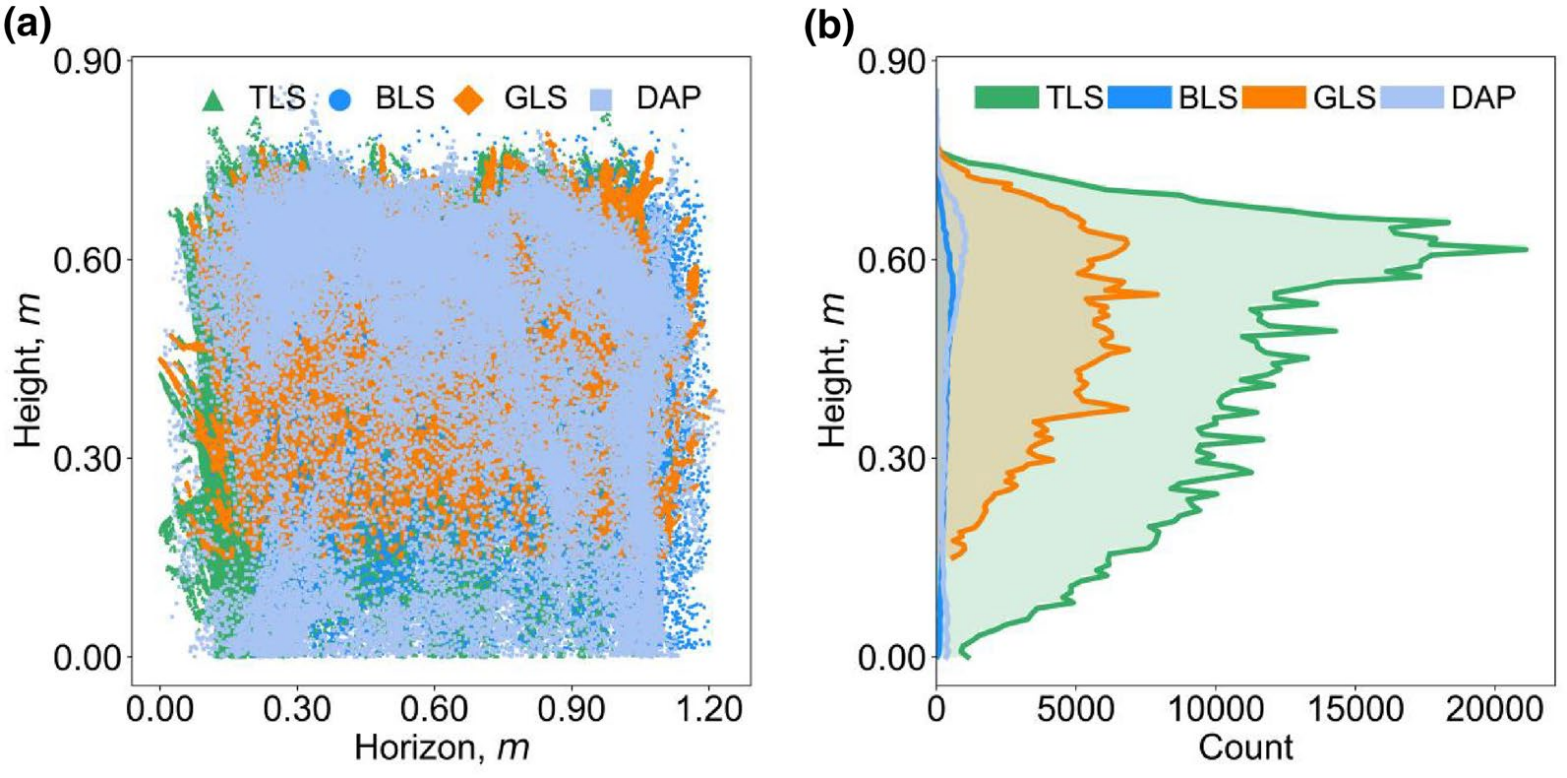
**a** Front view and **b** frequency distribution of points’ height value of TLS, BLS, GLS, and DAP data in the same plot

Although the best height quantiles are similar, the influences of height quantile selection on height estimation are different. DAP was easy to lose small targets such as the leaf tips of the canopy [46]. Meanwhile, DAP was difficult to capture the internal structure of the canopy [13], which leads to sparse point density (Fig. 10) and may illustrate why DAP-predicted CH accuracy was more sensitive to height quantiles (Fig. 4) and had a relative lager RMSE with other sensing datasets (RMSE > 0.05 m, Fig. 6b, c, f). By contrast, TLS, BLS, and GLS can generate high-density point clouds, enabling the characterization of inner canopy structure (Fig. 10). This may illustrate why GLS are less sensitive to the selection of height quantities, so are TLS and BLS (Fig. 4). Additionally, the GLS system used in this study may lose points near the ground due to the filtering method (Fig. 10b), which illustrated the overall underestimation and relative high bias of GLS-predicted data (Fig. 5c, Fig. 6d-f, Fig. 7c, Fig. 8c). However, it had a slight influence on the overall trend of CH assessment and RMSE (r = 0.82, Fig. 5c). Notably, despite the high point resolution of GLS, its ranging extent is much closer, making it easier to be saturated when predicting higher canopies, which can be seen if all the plots are used for height estimation in this study (Additional file 1: Fig. S6). This suggests that the choice of laser ranging extent is as important as the sensor resolution for high-precision crop phenotyping.

In conclusion, selecting the optimal height quantiles is critical in the evaluation of CH. Despite subtle differences, these best height metrics were very close in performance. Considering the more diverse datasets used in this study than in previous studies [38, 39, 43], the systematic evaluation of 3D sensing methods were unprecedented, which lays reliable foundations for the further cross-comparisons.

### CH estimation under various height groups, LAI groups, and GS groups

The CH estimate accuracies will obviously decrease when evaluated at CH subgroups (Fig. 7). This has been rarely reported in agriculture, but some similar findings have been drawn in forest CH estimation [31, 71]. The subgroup of lower CH plots (e.g., CH1) showed higher correlations (Table 3), which are consistent with previous studies that indicated the uncertainty of CH assessment by 3D sensing increased with height [59]. This may attribute to the increasing canopy complexity (e.g., crop canopy cover and plant density) with height [4, 15]. Meanwhile, canopy senescence and logging may also influence height estimation accuracy at high-height groups.

**Fig. 11:**
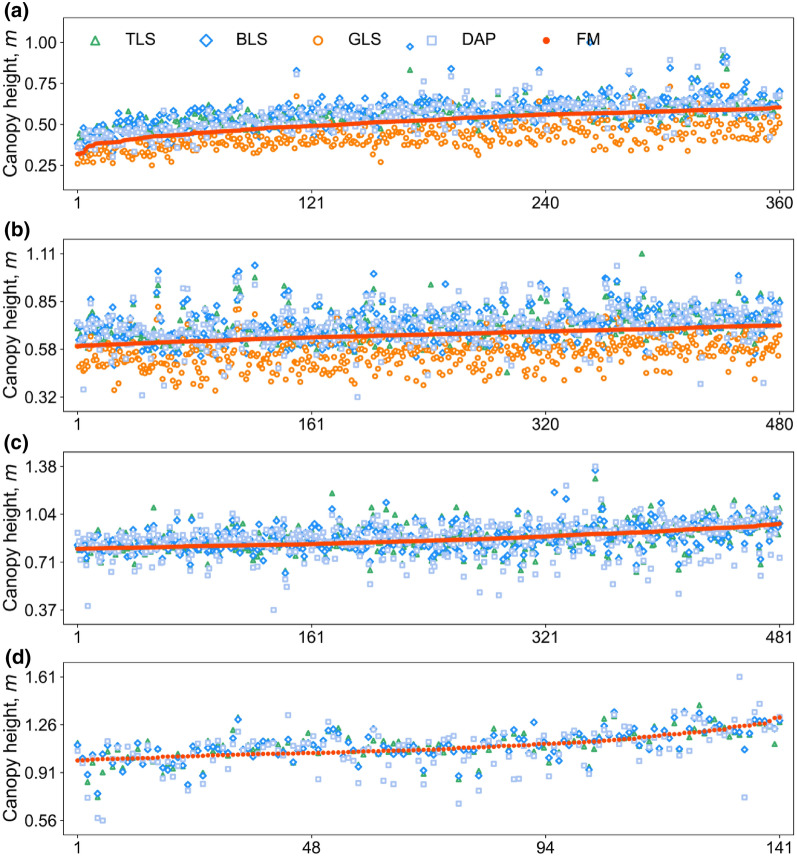
Canopy height (CH) observations from TLS (in the green triangle), BLS (in the blue rhombus), GLS (in the orange circle), DAP (in the blue light square), and FM (in the orange-red point) for **a** CH1, **b** CH2, **c** CH3, and **d** CH4 group. The x-axis represents the sorting order of field plots, and the y-axis represents the value of canopy height

This study found the TLS, BLS, and DAP showed overestimation in low CH groups (i.e., CH1 and CH2 groups) but are closer to field measurement in CH3 and CH4 groups (Figs. 7, 11). The possible reason is the canopy surface is not closed and looks uneven at the early stage. In this case, field measurement was hard to capture the highest CHs (observation) while the sensor measured height is the globally ranked height quantities (real max. height) of a plot. Although GLS had systematic underestimation due to its limited ranging extent, it had a better fitting effect with TLS and BLS (Additional file 1: Fig. S3), demonstrating the high reliability of ranging precision of 3D sensing technologies under different canopy structures. It is also the high precision of the GLS system (Table 1) that may illustrate why GLS keeps a low RMSE (0.04 m—0.05 m) with other 3D sensing datasets (Fig. 6d–f).

In addition, DAP-estimated height showed lower correlations with other 3D sensing datasets (Additional file 1: Fig. S3). This may be caused by the relatively lower data quality of the DAP point cloud. DAP point cloud was reconstructed from images, which are sensitive to environmental illumination, image quality, and reconstruction algorithms [2, 12, 16]. Some studies have demonstrated that the DAP has comparable accuracy with LiDAR in monitoring canopy height [10]. In this study, we further proved that DAP showed similar better results with LiDAR in field plots with lower CH (e.g., CH1), and found the accuracy would decrease at higher CH groups (Fig. 7). The decreasing accuracy may be caused by the large variations of estimated height at large CH groups where canopy structures are denser and complicated (Fig. 11).

By contrast, the CH estimate accuracies did not show an obvious decrease when evaluated at LAI or GS groups (Figs. 8 and 9). The possible reasons are the height range of data within each LAI or GS subgroup was relatively large. However, the accuracy at high LAI or late GS was also relatively lower, which may attribute to the more complex canopy structure [18, 45].

### Outlier analysis of different datasets

**Fig. 12:**
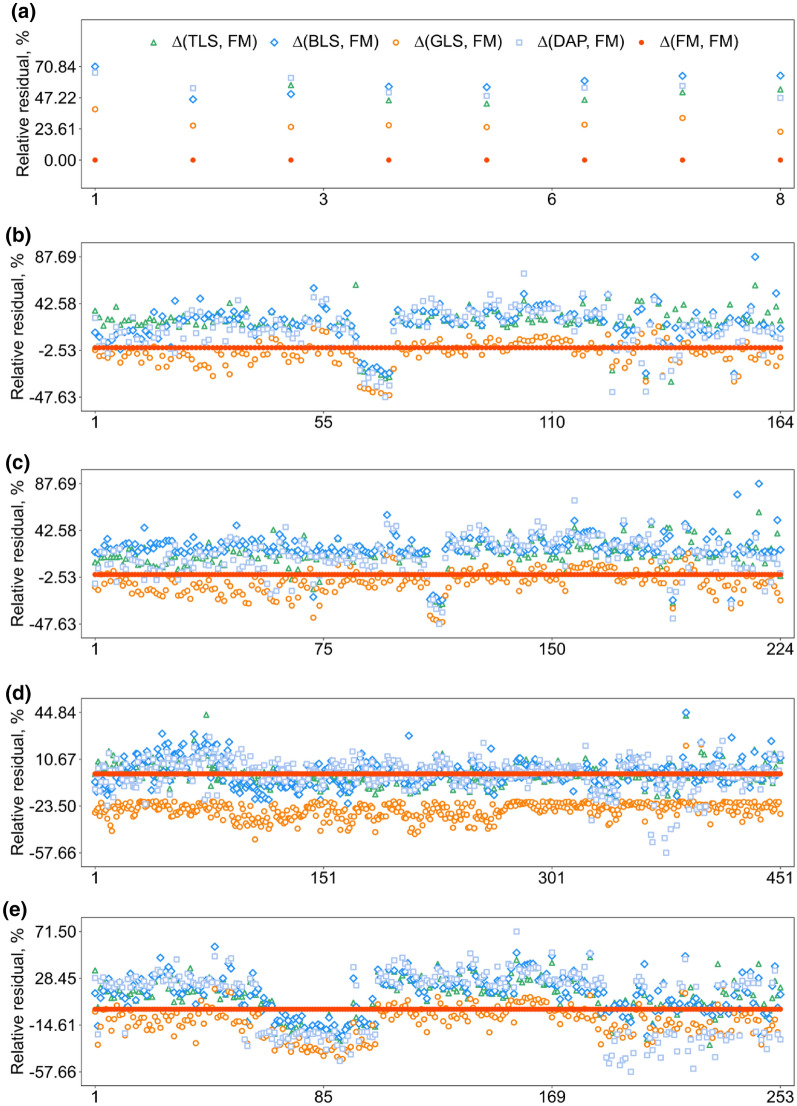
Relative canopy height residuals between 3D sensing-derived canopy height (CH) and FM. Suspicious results existed in **a** FM, **b** TLS-derived CH, **c** BLS-derived CH, **d** GLS-derived CH, and **e** DAP-derived CH according to Eq. 12–16. The x-axis represents the ID of field plots, and the y-axis represents the value of relative residuals. The green triangle, blue rhombus, orange circle, light blue square, and orange-red point represent the relative residuals of ∆(TLS, FM), ∆(BLS, FM), ∆(GLS, FM), ∆(DAP, FM), and ∆(FM, FM) in each subplot. Among them, the ∆(FM, FM) value is zero, which looks like a horizontal reference line in red

Error source analysis revealed that 8 plots existed FM error according to our definitions in Sect. "Error source analysis" (Fig. 12a). In these plots, heights estimated from all 3D sensing methods were 20% greater than FM, and the heights between different 3D sensing methods were closer. This indicated that FM may be inaccurate. By contrast, there are more potential suspicious CH results estimated from GLS (451), DAP (253), BLS (224), and TLS (164) (Fig. 12). Reasons for why the number of suspicious FM is fewer than other sensors may attribute to the strict judging conditions in Eq. 12. A FM value is suspicious only when it is suspicious to TLS, BLS, GLS, and DAP at the same time. In other words, if a FM is suspected as long as there are more than two suspects in the four kinds of comparisons (FM v.s. TLS, FM v.s. BLS, FM v.s. GLS, FM v.s. DAP), then the number of suspicious FMs will be more (Fig. 12).

In fact, there should be more errors coming from FM. For example, Fig. 12b shows the error source case of TLS, but it can be easily found that most TLS measurements were very consistent with BLS and DAP. This may imply FM and GLS are both suspicious, instead of TLS. Similar more suspicious cases of FM can be found in Fig. 12c–e. Although the overall underestimation of GLS data brought challenges for the above outlier analysis, the general trends still exist. As for the outlier estimations of GLS, most relative residuals were below −20% (Fig. 12d), which was mainly caused by the lack of ground point (Fig. 10b), indicating the importance of ground filtering in CH estimation.

### Field-measured canopy height may not be as accurate as believed

Our results showed that the height correlations between different 3D sensing (*r* = 0.87–0.97) are much better than the correlations between 3D sensing and FM (*r* = 0.82–0.89). The reasons may be two aspects. On the one hand, LiDAR and DAP are both accurate surveying and mapping technologies, they have good repeatability and consistency despite a wide variety of sensors and platforms. The TLS, BLS, and GLS systems with centimeter and millimeter resolutions have been proven accurate for estimating not only height but also other 3D traits [68, 76]. On the other hand, FM may be suspicious because it is based on subjective samples and is easily influenced by the terrain and other factors [1]. Some studies have also indicated that LiDAR may be more accurate than manual inspection [44].

Heritability quantifies the repeatability of the canopy height trait estimation, which is another prospect to evaluate the reliability of phenotyping methods and their potential for the breeding program [52]. On the one hand, the differences in the heritability of different data reflect their ability to characterize the subtle differences of CH among different varieties, as mentioned by Volpato et al. [67]. Our results proved that *H*^*2*^ of 3D sensing, especially* H*^*2*^ of LiDAR-derived CH, was larger than that derived from FM (Table 6), which may be determined by the higher accuracy of LiDAR systems. Higher H^2^ of the advanced 3D sensing tools indicate that they will facilitate better trait extraction for breeding. On the other hand, the overall heritability in the later growth period decreased, which may attribute to the prominent environmental impact of nitrogen treatment in the later growth period (e.g., logging). The environmental effects on the heritability of LiDAR-derived plant height have been proved by Madec et al. [43]. The dynamic change of *H*^*2*^ would be interested by plant breeders for selecting the right time to study the genotypic and/or environmental influences on phenotype [67].

### Contributions and implications

This study systematically evaluated the accuracy of CH estimation from advanced 3D sensing systems (TLS, BLS, GLS, and DAP) and FM using wheat plots of different varieties, fertilization levels, and growth stages. To our knowledge, this is the first effort that uses multiple 3D sensing technologies to evaluate their reliability for estimating CH with regard to different CH, LAI, and GS groups. Moreover, we analyzed the heritability from 3D sensing datasets and FM, proving the potential advantages of 3D sensing technologies in crop breeding.

However, there are still some interesting and important directions that need to be explored in the future. First, it is meaningful to deeply analyze the effect of operating modes of different 3D sensing technologies on CH monitoring. As for TLS, the scanning location settings (e.g., positions and total numbers) is important for acquiring a high-quality (higher density and less occlusion) point cloud [14, 68]. Although some pioneer studies have been conducted in forestry [74], it is still needed to have a scientific workflow of TLS in agriculture to ensure not only high accuracy but also improve efficiency. BLS is an economically friendly and easy-to-use platform. Designing the routine is critical and it has been discussed by Su et al. [58]. GLS is a kind of emerging phenotyping platform**,** which is mainly designed for crop phenotyping and has less been explored. This study highlights the necessity to integrate suitable sensors (e.g., longer-ranging ability) for different crop types, provide access to raw data, and enable more intelligent custom algorithms (e.g., filtering algorithm) for accurate phenotype extraction [29]. DAP is a low-cost system that has been widely used in phenotyping. However, the point cloud quality generated from DAP is affected by parameters such as sensor quality, camera shooting angle, routine overlap, and flight speed. This study determined the optimal flight height by a preliminary comparison experiment (Additional file 1: Fig. S1). More parameter comparison studies are worth exploring and can refer to Hu et al. [20]. Additionally, considering the UAV-LiDAR systems are more expensive than DAP and do not have obvious advantages in data quality [76], this study did not compare the UAV-LiDAR systems. However, we believe UAV-LiDAR systems are getting cheaper and the data quality is a good complement to DAP due to its higher penetration ability and robustness to light environments.

**Fig. 13:**
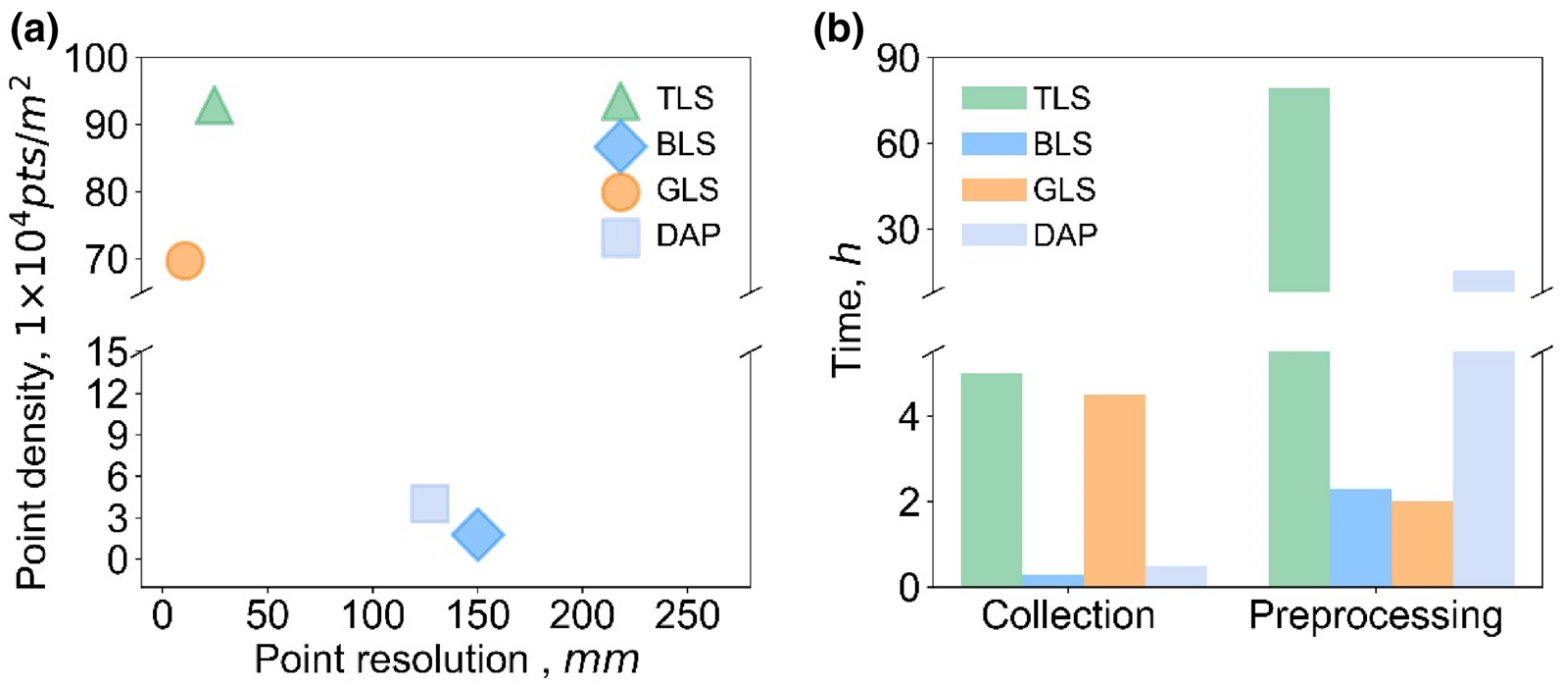
**a** Point accuracy and point density and **b** collection time and processing time from TLS (in the green triangle), BLS (in the blue rhombus), GLS (in the orange circle), and DAP (in the blue light square)

Secondly, the tradeoff between precision and efficiency is worth studying. Generally, data precision was depicted by point density and resolution. High point density usually has a high resolution (Fig. 13a). The possible reason why TLS has a higher point density but a lower resolution is the multi-scan registration [37]. More importantly, this study highlights that higher precision always needs a longer collection time, but does not mean more processing time (e.g., GLS) (Fig. 13). Among them, TLS has the longest data acquisition and processing time, because the reference targets and scanner need to be laboriously laid out during the scanning, and multi-scan data registration is time-consuming during reprocessing [7]. BLS has the shortest time (collection plus preprocessing), implicating this type of mobile mapping technology is worth promoting in the future, especially as cost decreases and accuracy increases. GLS not only has the highest point resolution but also has the shortest preprocessing time and affordable collection time, which benefits from the automatic data collection system and processing software [36]. However, this kind of phenotyping platform is still too expensive (Table 2). DAP has high collection efficiency, but the data quality is relatively low. Besides, the processing time of DAP is long not only caused by 3D reconstruction but also attribute to the manual de-noising process due to the low signal-to-noise ratio of the DAP point cloud. These preliminary explorations are of great significance for further in-depth and systematic analysis of cost and efficiency and the formulation of appropriate phenotypic working plans.

Finally, there is no standard for grouping CH and LAI. This study mainly divided 1920 plots into four different groups based on the value extent (maximum minus minimum) and frequency distribution. Although there are small differences in the spacing of the groupings and the number of groups is not exactly equal, the total sample sizes (i.e., 1920 plots) are unprecedented. The influence of CH, LAI, and GS on height measurement accuracy and heritability has been analyzed, but more quantitative evaluations are worth exploring, such as the specific CH and LAI thresholds for selecting the optimal measuring methods. Moreover, this study mainly studied the important CH trait in wheat, while more biologically meaningful and heritable traits in more crop types need further evaluation [35, 78].

## Conclusion

The study demonstrated novel insights into the accuracy and heritability of CH from 3D sensing and field measurement. Cross-comparisons among different sensor datasets showed higher correlations (r = 0.87 to 0.97) than comparisons with FM (r = 0.82 to 0.89). The correlation coefficients of CHs derived from 3D sensing and FM decreased obviously when evaluated with respect to different subgroups (CH, LAI, and GS), especially different CH subgroups. TLS and BLS were more reliable in monitoring CH under different subgroups according to their cross-comparisons and comparisons with FM. The outlier analysis found cases where FM may be error-prone. Moreover, 3D sensing methods showed even higher heritability than FM. Further studies about the best configurations of sensors and working plans are needed, the tradeoff between data quality and efficiency is worth exploring, and more traits deserve future efforts. These novel findings may give insights into the selection of advanced 3D sensing platforms for crop monitoring and may shed new light on the high-quality development of crop sciences (e.g. providing higher heritable traits for breeding).

## Supplementary Information

**Additional file 1: Fig. S1.** Canopy height correlations between the field measurement and DAP estimates at different flight heights. (a), (b), (c), and (d) are the results of flight height at 10, 20, 30, and 40m, respectively. The solid line represents the fitted line, and the dashed line represents the 1:1 line. The color bar shows the kernel density value of the point distribution, and the green to yellow represents the increase in kernel density. **Fig. S2.** (a) RMSE, (b) RMSE%, (c) Bias, and (d) Bias% between field measured height (FM) and different height quantiles (Hmax and H99) derived from the different 3D point cloud, including TLS, BLS, GLS, and DAP. The green triangle, blue diamond, dark orange circle, and baby blue square represent TLS vs. FM, BLS vs. FM, GLS vs. FM, and DAP vs. FM, respectively. **Fig. S3.** Correlations of cross-comparisons between different 3D sensing data estimated canopy height (CH) at four CH subgroups. (a), (b), (c), (d), (e), and (f) are comparisons of TLS vs. BLS, BLS vs. DAP, DAP vs. TLS, TLS vs. GLS, BLS vs. GLS, and DAP vs. GLS, respectively. The green triangle, blue diamond, orange circle, and light blue square represent the CH1, CH2, CH3, and CH4 groups, respectively. The solid line represents the fitted line, and the dashed line represents the 1:1 reference. **Fig. S4.** Correlations of cross-comparisons between different 3D sensing data estimated canopy height (CH) at four leaf area index (LAI) groups. (a), (b), (c), (d), (e), and (f) are comparisons of TLS vs. BLS, BLS vs. DAP, DAP vs. TLS, TLS vs. GLS, BLS vs. GLS, and DAP vs. GLS, respectively. The green triangle, blue diamond, orange circle, and light blue square represent the LAI1, LAI2, LAI3, and LAI4 groups, respectively. The solid line represents the fitted line, and the dashed line represents the 1:1 reference line. **Fig. S5.** Correlations of cross-comparisons between different 3D sensing data estimated canopy height (CH) at four growth stages (GS) groups. (a), (b), (c), (d), (e), and (f) are comparisons of TLS vs. BLS, BLS vs. DAP, DAP vs. TLS, TLS vs. GLS, BLS vs. GLS, and DAP vs. GLS, respectively. The green triangle, blue diamond, orange circle, and light blue square represent the jointing, heading, flowering, and maturity stages, respectively. The solid line represents the fitted line, and the dashed line represents the 1:1 reference line. **Fig. S6.** Correlations between GLS predicted datasets (including the measured canopy height over 0.82m) and other datasets. (a), (b), (c), and (d) respectively represent GLS vs. FM, TLS vs. GLS, BLS vs. GLS, and DAP vs. GLS. The solid line represents the fitted line, and the dashed line represents the 1:1 line. The color bar shows the kernel density value of the point distribution, and the green to yellow represents the increase in kernel density. **Table S1.** The performance of canopy height measurement with TLS, BLS, GLS, and DAP systems. R^2^, r, and RMSE are the coefficient of determination, correlation coefficient, and root mean square error, respectively. – represents the performance metric that is not available.

## Data Availability

The data that supports the findings of this study are available on request due to its large volume.
