# Supplementary material for: Field-measured canopy height may not be as accurate and heritable as believed: evidence from advanced 3D sensing
Source: Plant Methods. 2023 Apr 2;19:39. doi: 10.1186/s13007-023-01012-2 (PMC10069135; doi:10.1186/s13007-023-01012-2)
Supplement: Supplementary file 1 — Additional file 1: Fig. S1. Canopy height correlations between the field measurement and DAP estimates at different flight heights. (a), (b), (c), and (d) are the results of flight height at 10, 20, 30, and 40m, respectively. The solid line represents the fitted line, and the dashed line represents the 1:1 line. The color bar shows the kernel density value of the point distribution, and the green to yellow represents the increase in kernel density. Fig. S2. (a) RMSE, (b) RMSE%, (c) Bias, and (d) Bias% between field measured height (FM) and different height quantiles (Hmax and H99) derived from the different 3D point cloud, including TLS, BLS, GLS, and DAP. The green triangle, blue diamond, dark orange circle, and baby blue square represent TLS vs. FM, BLS vs. FM, GLS vs. FM, and DAP vs. FM, respectively. Fig. S3. Correlations of cross-comparisons between different 3D sensing data estimated canopy height (CH) at four CH subgroups. (a), (b), (c), (d), (e), and (f) are comparisons of TLS vs. BLS, BLS vs. DAP, DAP vs. TLS, TLS vs. GLS, BLS vs. GLS, and DAP vs. GLS, respectively. The green triangle, blue diamond, orange circle, and light blue square represent the CH1, CH2, CH3, and CH4 groups, respectively. The solid line represents the fitted line, and the dashed line represents the 1:1 reference. Fig. S4. Correlations of cross-comparisons between different 3D sensing data estimated canopy height (CH) at four leaf area index (LAI) groups. (a), (b), (c), (d), (e), and (f) are comparisons of TLS vs. BLS, BLS vs. DAP, DAP vs. TLS, TLS vs. GLS, BLS vs. GLS, and DAP vs. GLS, respectively. The green triangle, blue diamond, orange circle, and light blue square represent the LAI1, LAI2, LAI3, and LAI4 groups, respectively. The solid line represents the fitted line, and the dashed line represents the 1:1 reference line. Fig. S5. Correlations of cross-comparisons between different 3D sensing data estimated canopy height (CH) at four growth stages (GS) groups. (a), (b), (c), (d), [file 13007_2023_1012_MOESM1_ESM.docx]

**Supplementary files**


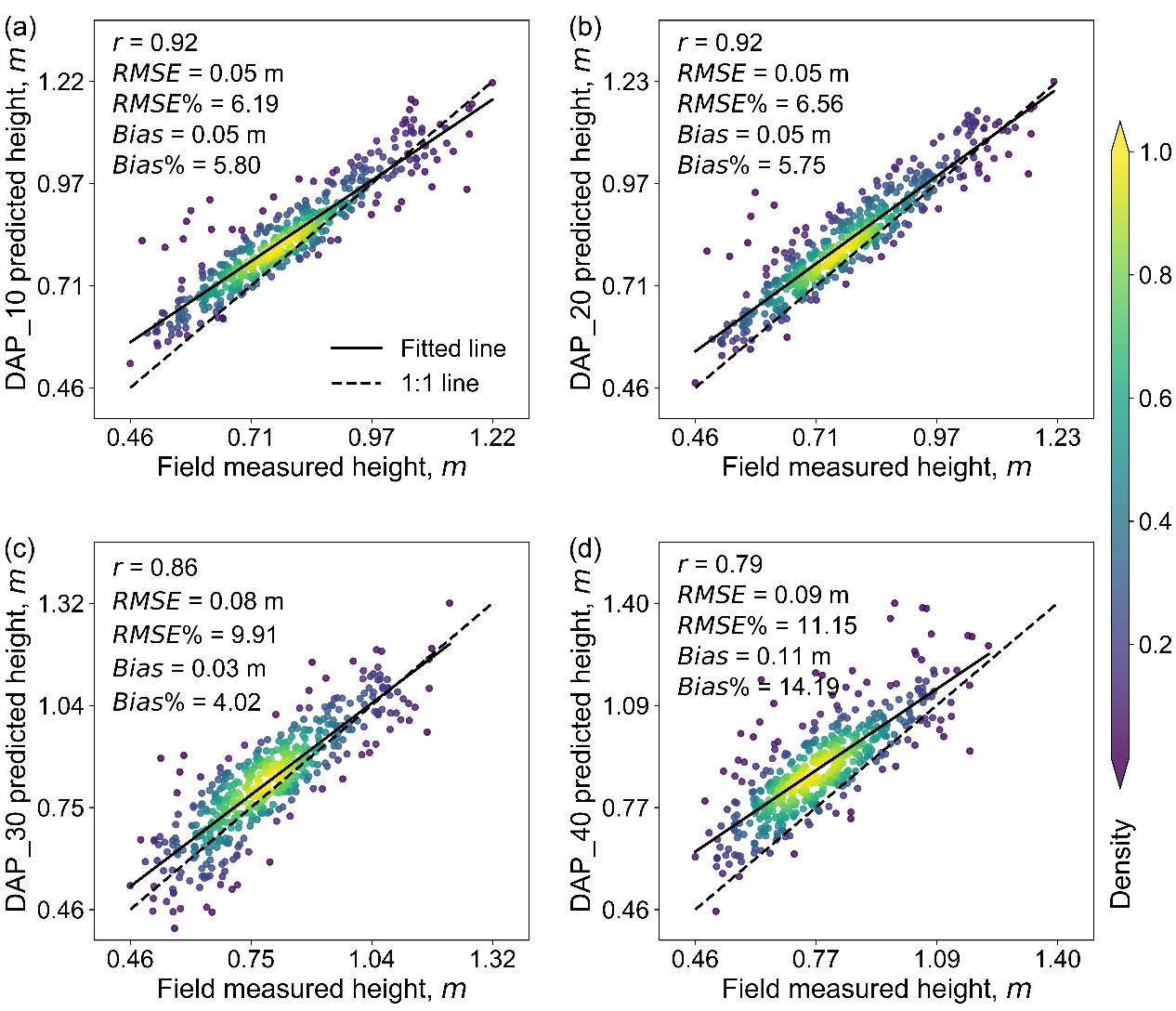


# Fig. S1 Canopy height correlations between the field measurement and DAP estimates at different flight heights. (a), (b), (c), and (d) are the results of flight height at 10, 20, 30, and 40m, respectively. The solid line represents the fitted line, and the dashed line represents the 1:1 line. The color bar shows the kernel density value of the point distribution, and the green to yellow represents the increase in kernel density.


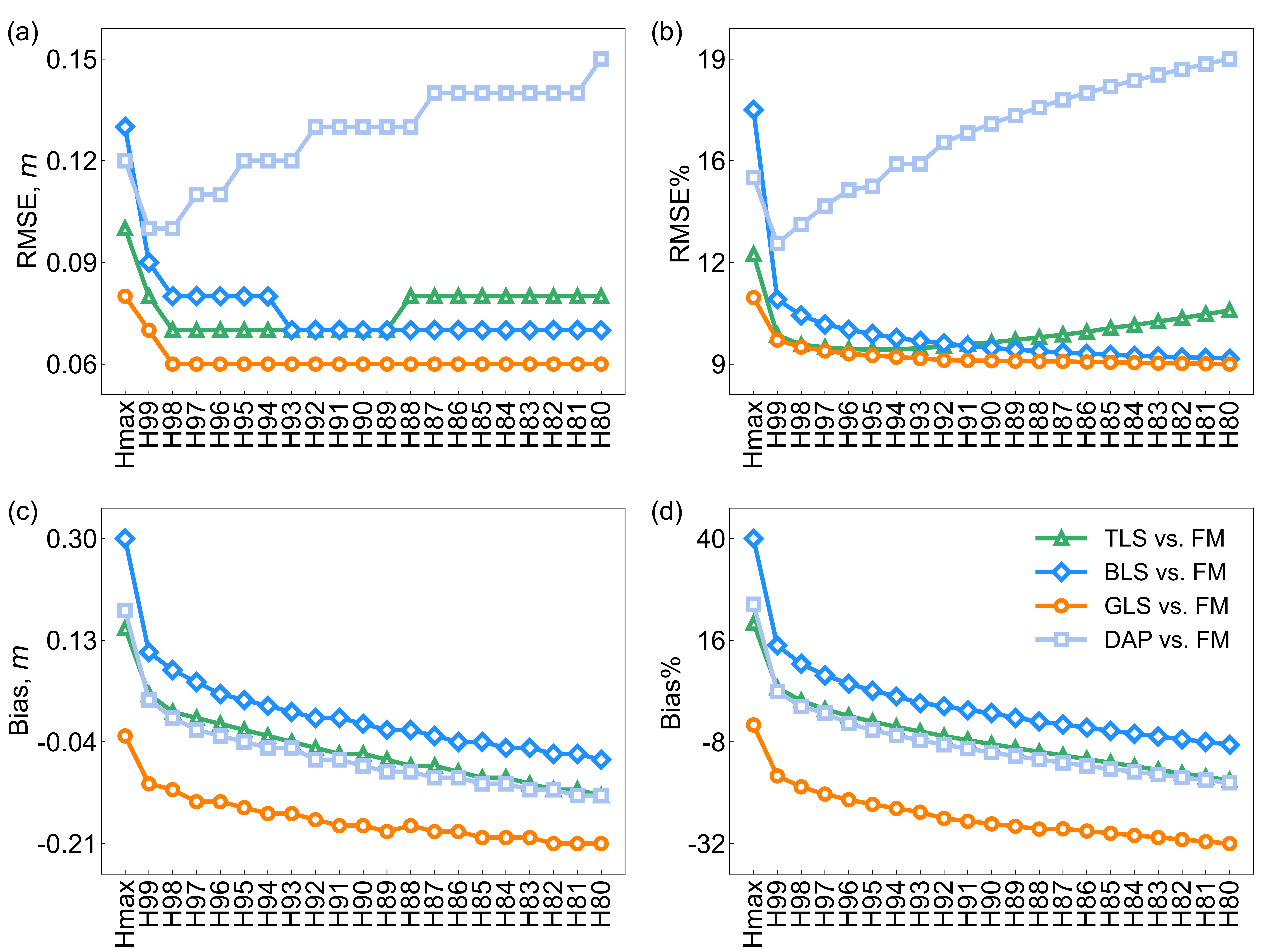


# Fig. S2 (a) RMSE, (b) RMSE%, (c) Bias, and (d) Bias% between field measured height (FM) and different height quantiles (Hmax and H99) derived from the different 3D point cloud, including TLS, BLS, GLS, and DAP. The green triangle, blue diamond, dark orange circle, and baby blue square represent TLS vs. FM, BLS vs. FM, GLS vs. FM, and DAP vs. FM, respectively.


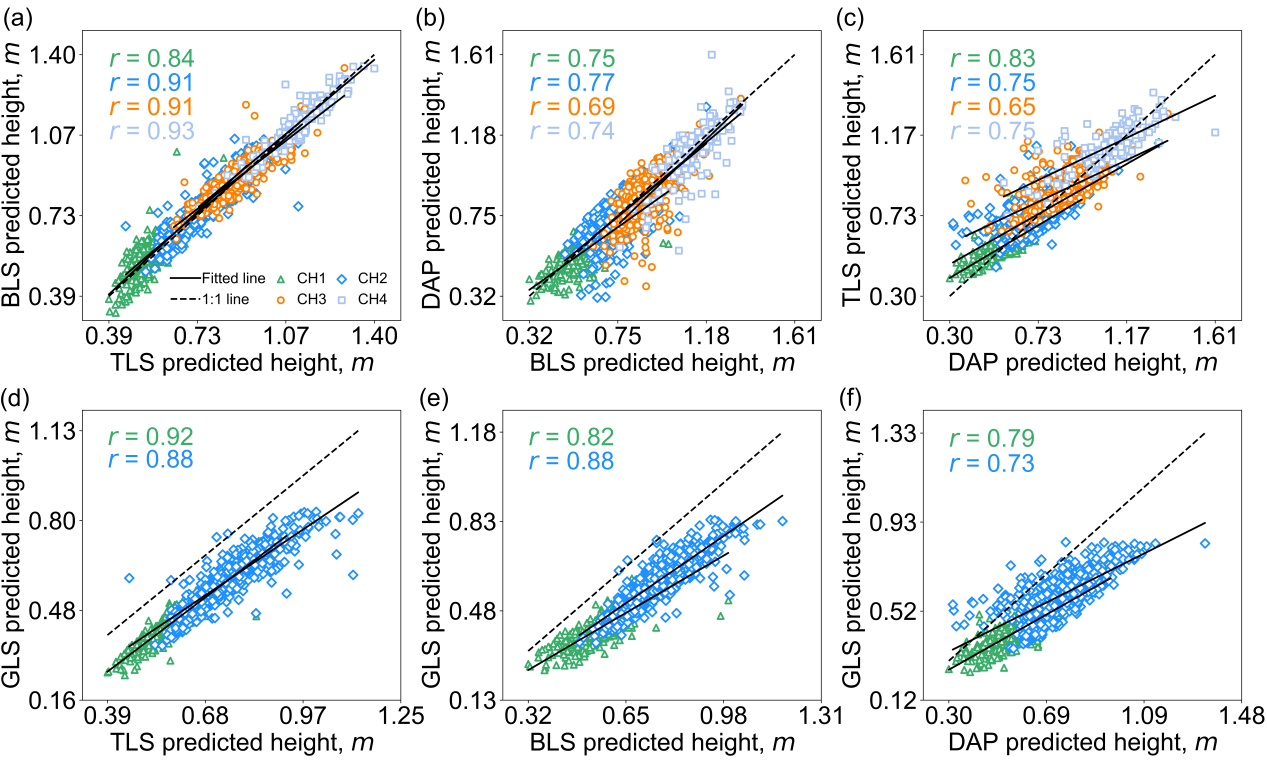


# Fig. S3 Correlations of cross-comparisons between different 3D sensing data estimated canopy height (CH) at four CH subgroups. (a), (b), (c), (d), (e), and (f) are comparisons of TLS vs. BLS, BLS vs. DAP, DAP vs. TLS, TLS vs. GLS, BLS vs. GLS, and DAP vs. GLS, respectively. The green triangle, blue diamond, orange circle, and light blue square represent the CH1, CH2, CH3, and CH4 groups, respectively. The solid line represents the fitted line, and the dashed line represents the 1:1 reference.


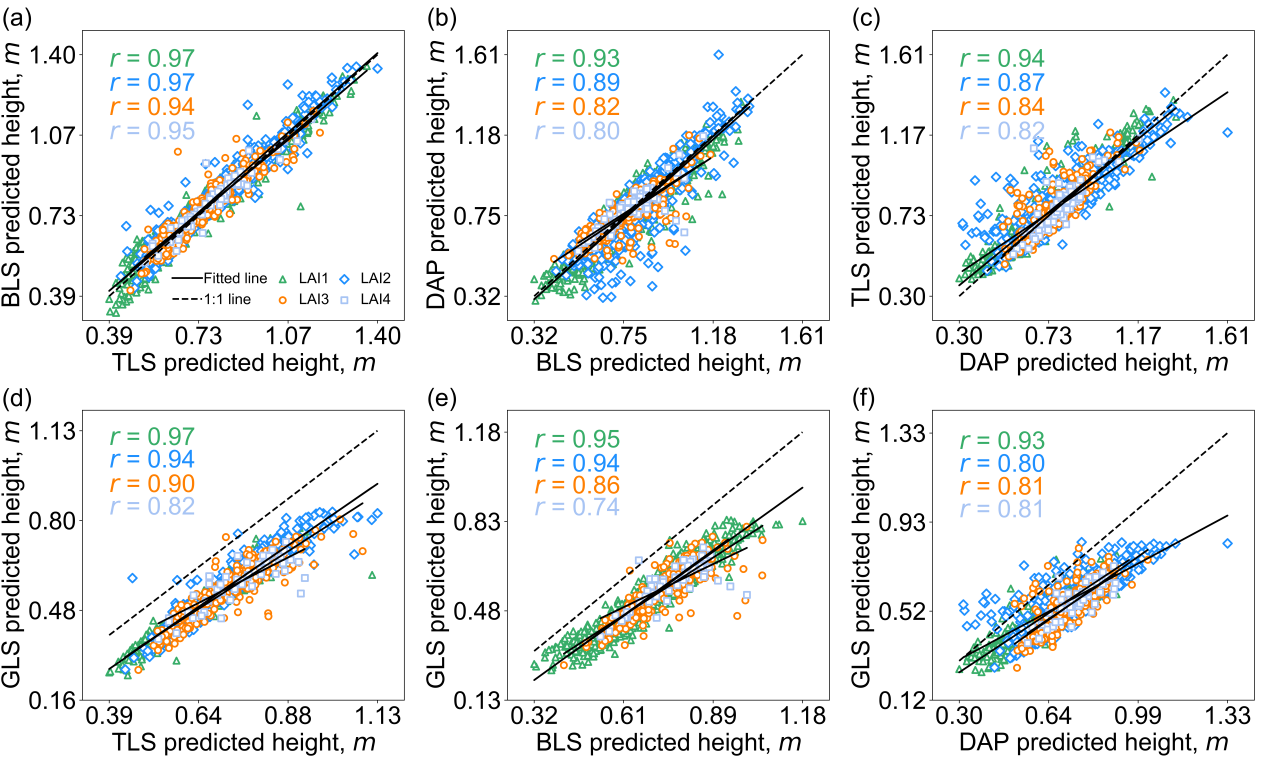


# Fig. S4 Correlations of cross-comparisons between different 3D sensing data estimated canopy height (CH) at four leaf area index (LAI) groups. (a), (b), (c), (d), (e), and (f) are comparisons of TLS vs. BLS, BLS vs. DAP, DAP vs. TLS, TLS vs. GLS, BLS vs. GLS, and DAP vs. GLS, respectively. The green triangle, blue diamond, orange circle, and light blue square represent the LAI1, LAI2, LAI3, and LAI4 groups, respectively. The solid line represents the fitted line, and the dashed line represents the 1:1 reference line.


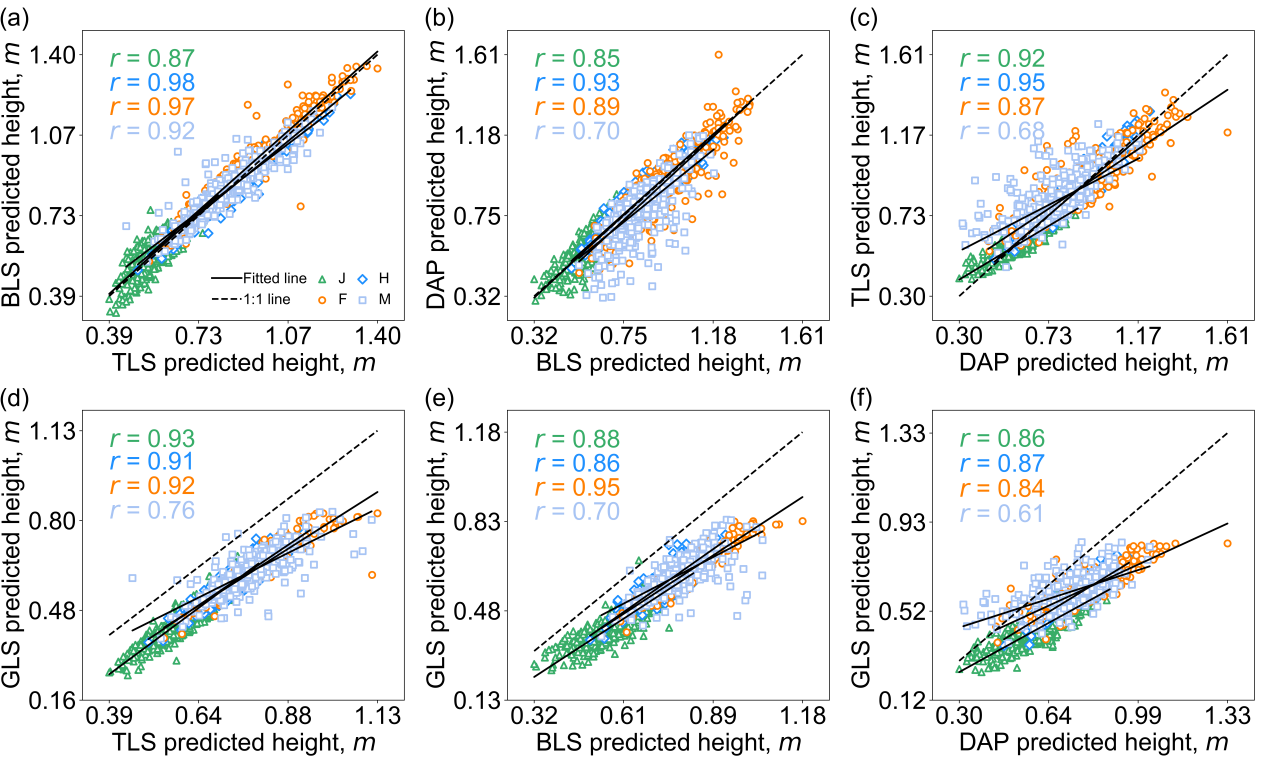


# Fig. S5 Correlations of cross-comparisons between different 3D sensing data estimated canopy height (CH) at four growth stages (GS) groups. (a), (b), (c), (d), (e), and (f) are comparisons of TLS vs. BLS, BLS vs. DAP, DAP vs. TLS, TLS vs. GLS, BLS vs. GLS, and DAP vs. GLS, respectively. The green triangle, blue diamond, orange circle, and light blue square represent the jointing, heading, flowering, and maturity stages, respectively. The solid line represents the fitted line, and the dashed line represents the 1:1 reference line.


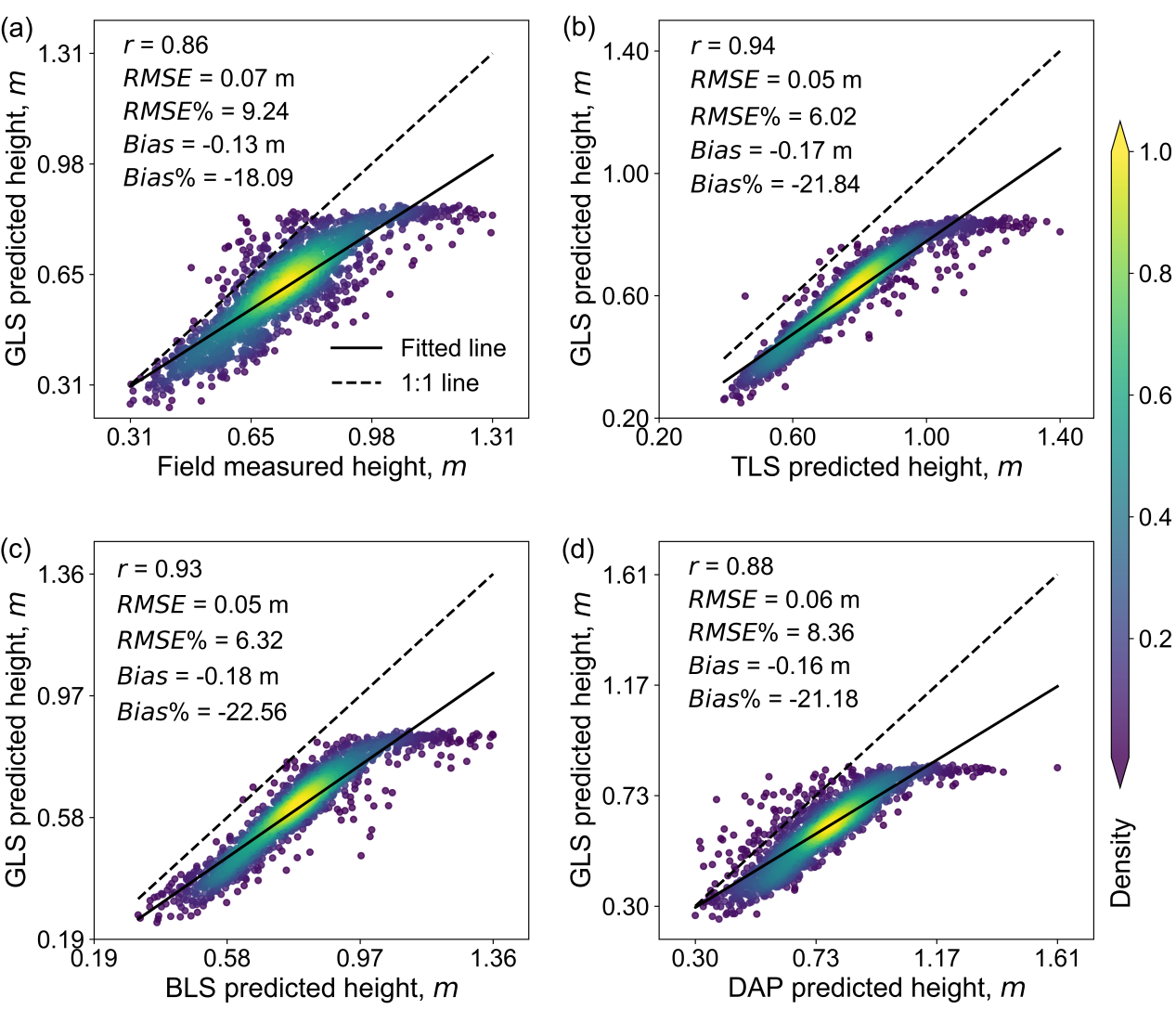


# Fig. S6 Correlations between GLS predicted datasets (including the measured canopy height over 0.82m) and other datasets. (a), (b), (c), and (d) respectively represent GLS vs. FM, TLS vs. GLS, BLS vs. GLS, and DAP vs. GLS. The solid line represents the fitted line, and the dashed line represents the 1:1 line. The color bar shows the kernel density value of the point distribution, and the green to yellow represents the increase in kernel density.

# Table S1. The performance of canopy height measurement with TLS, BLS, GLS, and DAP systems. *R^2^*, *r, and RMSE* are the coefficient of determination, correlation coefficient, and root mean square error, respectively. – represents the performance metric that is not available.

| 3D sensing systems | Plant types | Performances | References |
| --- | --- | --- | --- |

| TLS | Maize | R^2^=0.93 | (Tilly et al., 2014b) |
| --- | --- | --- | --- |
|  | Wheat | R^2^=0.97 | (Guo et al., 2019) |
|  | Cotton | R^2^=0.97 | (Sun et al., 2018) |
|  | Rice | R^2^=0.91 | (Tilly et al., 2014a) |
|  | Barley/ pea/ bean | R^2^=0.95/ R^2^=0.93/ R^2^=0.91 | (El-Naggar et al., 2021) |
| BLS | Wheat | R^2^=0.87 | (Zhu et al., 2021) |
|  | Tree | *RMSE*=1.66 m | (Ko et al., 2021) |
|  | Tree | R^2^=0.65 | (Su et al., 2021) |
| GLS | Wheat | - | (Li *et al.*, 2022) |
|  | Tree | - | (Sun *et al.*, 2022) |
| ULS | Wheat | R^2^=0.88 | (Hütt et al., 2022) |
|  | Tree | R^2^=0.99 | (Hartley et al., 2020) |
|  | Sugar beet/ wheat/ potato | R^2^=0.70/ R^2^=0.78/ R^2^=0.50 | (Jelle ten Harkel et al., 2019) |
| DAP | Maize/sorghum | R^2^=0.91/ R^2^=0.85 | (Malambo et al., 2018) |
|  | Wheat | R^2^=0.92 | (Hütt et al., 2022) |
|  | Tree | R^2^=0.94 | (Hartley et al., 2020) |
|  | Pea/ chickpea/ Winter canola/ Spring canola | *r*=0.71/ *r*=0.91/  *r*=0.86/ *r*=0.92 | (Zhang et al., 2021) |
|  | Corn | R^2^=0.78 | (Su et al., 2019) |
|  | Soybean | *r*=0.82 | (Bai et al., 2022) |
|  | Wheat | R^2^=0.77 | (Wang et al., 2022) |

**References**

Bai, D., Li, D., Zhao, C., Wang, Z., Shao, M., Guo, B., Liu, Y., Wang, Q., Li, J., Guo, S., Wang, R., Li, Y. H., Qiu, L. J. & Jin, X. (2022). Estimation of soybean yield parameters under lodging conditions using rgb information from unmanned aerial vehicles. *Front Plant Sci* 13: 1012293-1012312.

El-Naggar, A. G., Jolly, B., Hedley, C. B., Horne, D., Roudier, P. & Clothier, B. E. (2021). The use of terrestrial lidar to monitor crop growth and account for within-field variability of crop coefficients and water use. *Computers and Electronics in Agriculture* 190(6): 106416-106432.

Guo, T., Fang, Y., Cheng, T., Tian, Y., Zhu, Y., Chen, Q., Qiu, X. & Yao, X. (2019). Detection of wheat height using optimized multi-scan mode of lidar during the entire growth stages. *Computers and Electronics in Agriculture* 165(6): 104959-104968.

Hartley, R. J. L., Leonardo, E. M., Massam, P., Watt, M. S., Estarija, H. J., Wright, L., Melia, N. & Pearse, G. D. (2020). An assessment of high-density uav point clouds for the measurement of young forestry trials. *Remote Sensing* 12(24): 4039-4059.

Hütt, C., Bolten, A., Hüging, H. & Bareth, G. (2022). Uav lidar metrics for monitoring crop height, biomass and nitrogen uptake: A case study on a winter wheat field trial. *PFG – Journal of Photogrammetry, Remote Sensing and Geoinformation Science* 22(6): 2512-2819.

Jelle ten Harkel, J., Bartholomeus, H. & Kooistra, L. (2019). Biomass and crop height estimation of different crops using uav-based lidar. *Remote Sensing* 12(1): 17-35.

Ko, C., Lee, S., Yim, J., Kim, D. & Kang, J. (2021). Comparison of forest inventory methods at plot-level between a backpack personal laser scanning (bpls) and conventional equipment in jeju island, south korea. *Forests* 12(3): 308-321.

Li, Q., Jin, S., Zang, J., Wang, X., Sun, Z., Li, Z., Xu, S., Ma, Q., Su, Y., Guo, Q. & Jiang, D. (2022). Deciphering the contributions of spectral and structural data to wheat yield estimation from proximal sensing. *The Crop Journal* 11: 2214-2225.

Malambo, L., Popescu, S. C., Murray, S. C., Putman, E., Pugh, N. A., Horne, D. W., Richardson, G., Sheridan, R., Rooney, W. L., Avant, R., Vidrine, M., McCutchen, B., Baltensperger, D. & Bishop, M. (2018). Multitemporal field-based plant height estimation using 3d point clouds generated from small unmanned aerial systems high-resolution imagery. *International Journal of Applied Earth Observation and Geoinformation* 64: 31-42.

Su, W., Zhang, M., Bian, D., Liu, Z., Huang, J., Wang, W., Wu, J. & Guo, H. (2019). Phenotyping of corn plants using unmanned aerial vehicle (uav) images. *Remote Sensing* 11(17): 2021-2140.

Su, Y., Guo, Q., Jin, S., Guan, H., Sun, X., Ma, Q., Hu, T., Wang, R. & Li, Y. (2021). The development and evaluation of a backpack lidar system for accurate and efficient forest inventory. *IEEE Geoscience and Remote Sensing Letters* 18(9): 1660-1664.

Sun, S., Li, C., Paterson, A. H., Jiang, Y., Xu, R., Robertson, J. S., Snider, J. L. & Chee, P. W. (2018). In-field high throughput phenotyping and cotton plant growth analysis using lidar. *Front Plant Sci* 9(1): 16-33.

Sun, Z., Li, Q., Jin, S., Song, Y., Xu, S., Wang, X., Cai, J., Zhou, Q., Ge, Y., Zhang, R., Zang, J. & Jiang, D. (2022). Simultaneous prediction of wheat yield and grain protein content using multitask deep learning from time-series proximal sensing. *Plant Phenomics* 2022(3): 1-13.

Tilly, N., Hoffmeister, D., Cao, Q., Huang, S., Lenz-Wiedmann, V., Miao, Y. & Bareth, G. (2014a). Multitemporal crop surface models: Accurate plant height measurement and biomass estimation with terrestrial laser scanning in paddy rice. *Journal of Applied Remote Sensing* 8: 083671-0836693.

Tilly, N., Hoffmeister, D., Schiedung, H., Hütt, C., Brands, J. & Bareth, G. (2014b). Terrestrial laser scanning for plant height measurement and biomass estimation of maize. *The International Archives of the Photogrammetry, Remote Sensing and Spatial Information Sciences* XL-7: 181-187.

Wang, D., Li, R., Zhu, B., Liu, T., Sun, C. & Guo, W. (2022). Estimation of wheat plant height and biomass by combining uav imagery and elevation data. *Agriculture* 13(1): 9-30.

Zhang, C., Craine, W. A., McGee, R. J., Vandemark, G. J., Davis, J. B., Brown, J., Hulbert, S. H. & Sankaran, S. (2021). High‐throughput phenotyping of canopy height in cool‐season crops using sensing techniques. *Agronomy Journal* 113(4): 3269-3280.

Zhu, Y., Sun, G., Ding, G., Zhou, J., Wen, M., Jin, S., Zhao, Q., Colmer, J., Ding, Y., Ober, E. S. & Zhou, J. (2021). Large-scale field phenotyping using backpack lidar and cropquant-3d to measure structural variation in wheat. *Plant Physiol* 187(2): 716-738.
